# Toxicological Activities of *Pteridium aquilinum* Rhizomes and Fiddleheads in HPV16-Transgenic Mice

**DOI:** 10.3390/biology15120976

**Published:** 2026-06-22

**Authors:** Beatriz Medeiros-Fonseca, Ana I. Faustino-Rocha, Maria João Pires, Maria João Neuparth, Felisbina Queiroga, Isabel Gaivão, Marcelo D. Catarino, Susana M. Cardoso, Margarida M. Bastos, Luís Félix, Carlos Venâncio, Fernanda Seixas, Cármen Vasconcelos-Nóbrega, Helena Vala, Rui Medeiros, Paula A. Oliveira, Rui M. Gil da Costa

**Affiliations:** 1 Centre for the Research and Technology of Agro-Environmental and Biological Sciences (CITAB), University of Trás-os-Montes and Alto Douro (UTAD), 5000-801 Vila Real, Portugal; fonsecabeatriz@live.com.pt (B.M.-F.); anafaustino@uevora.pt (A.I.F.-R.); joaomp@utad.pt (M.J.P.); lfelix@utad.pt (L.F.); cvenanci@utad.pt (C.V.); cnobrega@esav.ipv.pt (C.V.-N.); hvala@esav.ipv.pt (H.V.); pamo@utad.pt (P.A.O.); 2 Institute for Innovation, Capacity Building and Sustainability of Agri-Food Production (Inov4Agro), University of Trás-os-Montes and Alto Douro (UTAD), 5000-801 Vila Real, Portugal; 3 Molecular Oncology and Viral Pathology Group, Research Center of IPO Porto (CI-IPOP)/RISE@CI-IPOP (Health Research Network), Portuguese Oncology Institute of Porto (IPO Porto)/Pathology and Laboratory Medicine Department/Clinical Pathology/Porto Comprehensive Cancer Center Raquel Seruca (Porto. CCC), 4200-072 Porto, Portugal; ruimedei@ipoporto.min-saude.pt; 4 Department of Zootechnics, School of Sciences and Technology, University of Évora, 7000-812 Évora, Portugal; 5 Comprehensive Health Research Center, University of Évora, 7000-812 Évora, Portugal; 6 Department of Veterinary Sciences, University of Trás-os-Montes and Alto Douro (UTAD), 5000-801 Vila Real, Portugal; fqueiroga@utad.pt; 7 Research Center in Physical Activity, Health and Leisure (CIAFEL), Laboratory for Integrative and Translational Research in Population Health (ITR), University of Porto (FADEUP), Faculty of Sport, 4200-450 Porto, Portugal; mneuparth@hotmail.com; 8 UCIBIO-Applied Molecular Biosciences Unit, Toxicologic Pathology Research Laboratory, University Institute of Health Sciences (1H-TOXRUN, IUCS-CESPU), 4585-116 Gandra, Portugal; 9 Animal and Veterinary Research Centre (CECAV), Associate Laboratory for Animal and Veterinary Sciences (AL4AnimalS), University of Trás-os-Montes and Alto Douro (UTAD), 5000-801 Vila Real, Portugal; igaivao@utad.pt (I.G.); fseixas@utad.pt (F.S.); 10 Centre for the Study of Animal Science, CECA-ICETA, University of Porto, 4200-427 Porto, Portugal; 11 Department of Genetics and Biotechnology, University of Trás-os-Montes and Alto Douro (UTAD), 5000-801 Vila Real, Portugal; 12 LAQV-REQUIMTE, Department of Chemistry, University of Aveiro, 3810-193 Aveiro, Portugal; mcatarino@ua.pt (M.D.C.); susanacardoso@ua.pt (S.M.C.); 13 Laboratory for Process Engineering, Environment, Biotechnology and Energy (LEPABE), Associate Laboratory in Chemical Engineering (ALiCE), Faculty of Engineering, University of Porto (FEUP), 4200-465 Porto, Portugal; mbastos@fe.up.pt; 14 Agrarian School of Viseu, Polytechnic Institute of Viseu, Quinta da Alagoa-Estrada de Nelas Ranhados, 3500-606 Viseu, Portugal; 15 CERNAS-IPV Research Centre, Instituto Politécnico de Viseu, Campus Politécnico, Repeses, 3504-510 Viseu, Portugal; 16 Research Department of the Portuguese League Against Cancer Regional Nucleus of the North (LPCC-NRN), 4200-177 Porto, Portugal; 17 Faculty of Medicine, University of Porto, 4200-319 Porto, Portugal; 18 Institute of Biomedical Sciences Abel Salazar (ICBAS), 4050-313 Porto, Portugal; 19 Biomedical Research Center (CEBIMED), Faculty of Health Sciences of Fernando Pessoa University (UFP), 4249-004 Porto, Portugal; 20 Department of Pathology, State University of Maranhão (UEMA), São Luís 65055-310, Brazil

**Keywords:** HPV16, *Pteridium aquilinum*, rhizomes, fiddleheads, mouse model

## Abstract

*Pteridium aquilinum* is a globally distributed plant species, highly adaptable to various environments and historically significant as a food source for both animals and humans. This study evaluated the in vivo effects of aqueous extracts from *Pteridium aquilinum* rhizomes and freeze-dried fiddleheads in a transgenic mouse model of human papillomavirus type 16 (HPV16)-induced cancer. Rhizome extract was administered in drinking water at concentrations of 0.0125, 0.025, and 0.05 g/mL for 28 days across six experimental groups (n = 5): G1 (wild-type, control), G2 (wild-type, 0.05 g/mL), G3 (HPV, control), G4 (HPV, 0.0125 g/mL), G5 (HPV, 0.025 g/mL), and G6 (HPV, 0.05 g/mL). Freeze-dried fiddleheads were incorporated into the diet at concentrations of 12.5%, 25%, and 50%, also using six groups (n = 5). Humane endpoints, body weight, and food and water consumption were monitored weekly. The rhizome extract was associated with more pronounced biological effects compared to fiddleheads, particularly at the histological and molecular levels. Conversely, freeze-dried fiddleheads were better tolerated. The results indicate that rhizomes were associated with great biological impact under the present experimental conditions, particularly in HPV16 transgenic mice, highlighting a potential synergistic effect with HPV. The potential risks associated with *Pteridium aquilinum* consumption, as well as preparation methods, should be carefully considered, even for rhizomes which are often regarded as less harmful than other plant parts.

## Simple Summary

*Pteridium aquilinum*, commonly known as bracken fern, is a plant found worldwide and has long been used as a food source for both animals and humans. However, some parts of this plant are known to contain harmful substances. In this study, we investigated the effects of extracts prepared from the underground stems (rhizomes) and from young shoots (fiddleheads) of bracken fern in mice, including animals carrying a virus associated with cancer development. The extracts were administered either in drinking water or mixed into the diet for four weeks, and the animals’ health, body weight, and food and water intake were carefully monitored. We found that extracts from the rhizomes were associated with more pronounced biological effects, particularly in the skin, liver, and spleen, and these effects were more evident in mice carrying the cancer-associated virus. In contrast, the fiddleheads were better tolerated. These findings demonstrate that different parts of the same plant can have distinct biological effects and that the way the plant is prepared and consumed is important. Overall, our results highlight potential health concerns associated with bracken fern consumption and emphasize the need for caution.

## 1. Introduction

*Pteridium aquilinum*, commonly known as bracken fern or common bracken, is a species of pteridophyte belonging to the family Dennstaedtiaceae [1]. It is worldwide and is mainly found in bush areas, thickets, and cultivated land [2]. It preferentially develops in acidic, deep soils and in humid, sometimes shaded environments [3,4]. This plant is used as a food source by several human populations, as well as by animals, and can exhibit significant toxic effects [2]. In the livestock industry, the ingestion of toxic plants such as bracken has a negative economic impact, as it directly affects animal production, including weight loss, reduced growth, and reproductive disorders, and is also associated with increased medical costs and changes in animal management [1,5]. In addition, its ingestion by animals can indirectly affect human health through the contamination of animal-derived food products, such as meat, milk, and derivatives with bracken toxins [6,7]. The toxic effects caused by this plant vary according to the animal species, the ingested concentration and the duration of exposure [2,5].

*Pteridium aquilinum* contains numerous compounds, including thiaminase, tannins, quercetin, shikimic acid, prunasin, kaempferol and illudane-type glycosides such as ptaquiloside [1]. Once ingested, it is difficult to determine the contribution of each constituent to the observed adverse effects, as these compounds possess different biochemical properties [1]. Ptaquiloside is considered the main carcinogenic agent, capable of inducing intestinal, mammary, and bladder tumors in rats [8,9,10]. This compound can be present up to 12.94 μg/g of dry plant material. However, its concentration and toxicity depend on the genotype of *Pteridium aquilinum* [11]. Although this toxin is present in all parts of the plant, its concentration varies depending on several factors, including the plant part, age and seasonality [12]. Thus, the fiddleheads have been reported to contain higher levels of ptaquiloside, whereas rhizome is generally considered to have lower carcinogenic activity [12,13].

Overall, a diet based on *Pteridium aquilinum* can cause several symptoms in animals, including neurological disorders associated with thiamine deficiency, mainly observed in horses; acute hemorrhagic disease in cattle and sheep; retinal atrophy in sheep and enzootic hematuria in cattle, associated with the development of bladder and digestive tract cancer [14]. The ingestion of illudane glycosides present in *Pteridium aquilinum* has been associated with carcinogenesis and is known to facilitate papillomavirus (PV) infection in animals [15]. These compounds may act not only as direct carcinogens but also as co-carcinogenic agents, enhancing the progression of pre-existing lesions [15].

In this context, the K14-HPV16 transgenic mouse model represents a particularly relevant system to investigate these interactions. These animals develop progressive epithelial alterations driven by HPV16 oncogene expression, including hyperplasia, dysplasia and carcinoma, accompanied by chronic inflammation and increased cellular proliferation [16]. HPV-driven cell proliferation and reduced DNA repair enhance tissue susceptibility to genotoxic compounds, such as those present in *Pteridium aquilinum*, thereby potentially amplifying their carcinogenic quality [17]. Previous studies have further demonstrated that bracken-derived compounds, such as ptaquiloside, can act as co-carcinogens in this model [18], supporting the relevance of this system to investigate interactions between viral and environmental factors.

Accordingly, the present study aims to investigate the in vivo effects of an infusion of aqueous extract from *Pteridium aquilinum* rhizomes and freeze-dried fiddleheads from *Pteridium aquilinum*, in a transgenic mouse model of HPV16-induced cancer [16,17,18].

## 2. Materials and Methods

### 2.1. Bracken Samples

Samples of *Pteridium aquilinum* rhizomes were collected in April 2022, and the fiddlehead samples were collected in May 2023, both from the same location in Serra das Meadas, Lamego, Portugal (41.0709872, −7.8613863). A bracken specimen was deposited in the herbarium of the University of Trás-os-Montes and Alto Douro (reference number 24270).

#### 2.1.1. Sample Treatment and Storage

The rhizomes were washed with distilled water and dried in an oven (HotCold260, Astori Tecnica s.r.l., Poncarale (BS), Italy) for five weeks at 35 °C. They were then stored in a cool, dry place, protected from light. Fiddleheads were washed with distilled water, dried with a sterile cloth, weighed, and stored at −80 °C before being freeze-dried at −55.5 °C for four days using a Christ Model Alpha 1-4 LSCplus freeze dryer (Martin Christ Gefriertrocknungsanlagen GmbH, Osterode am Harz, Germany). They were then macerated and stored in a cool, dry place.

#### 2.1.2. Sample Preparation

Rhizomes were prepared by hot-water extraction. Briefly, three concentrations (0.0125, 0.025, and 0.050 g/mL) of rhizome extract were prepared by adding the plant material to water heated to 90 °C for 5 min using a French press. The extracts were then filtered and allowed to cool at room temperature (20–25 °C) for 20 min. Freeze-dried fiddlehead samples, corresponding to a ratio of 0.16 g freeze-dried material per gram of fresh fiddlehead (g/g), were incorporated into a standard laboratory animal diet (4RF21, Mucedola, Italy) at three inclusion levels: 12.5%, 25%, and 50%.

The experimental design and dose selection were guided by previous toxicological data in order to establish biologically relevant exposure ranges for both assays. For the rhizome extract assay, the concentration range (0.050, 0.025, and 0.0125 g/mL) was derived from published acute toxicity data reporting a maximum tolerated exposure of 60 g/kg in mice for *Pteridium aquilinum* infusion (Duan, 2007) [19]. Given the lack of direct equivalence between in vivo dose (g/kg) and infusion concentration (g/mL), a serial dilution approach was adopted to generate a graded exposure range suitable for toxicological assessment. Similarly, for the freeze-dried fiddlehead assay, inclusion levels of 50%, 25%, and 12.5% were established based on ptaquiloside concentrations per kilogram of fresh biomass reported by Costa et al. (2020) [18]. In this case, a comparable serial dilution strategy was also applied to define a decreasing gradient of dietary exposure, ensuring comparability between conditions while remaining within exposure levels described in the literature.

#### 2.1.3. Chemical Composition

UHPLC-ESI-MS analysis was performed using an Ultimate 3000 (Dionex Co., San Jose, CA, USA) equipped with a 3000 Diode Array Detector (DAD) (Dionex Co., San Jose, CA, USA) and coupled to a Thermo LTQ XL (Thermo Scientific, San Jose, CA, USA) ion trap mass spectrometer with an ESI source. The rhizome extract was analyzed directly, while the fiddlehead samples were first extracted with 80% ethanol prior to analysis. Chomatographic separation was performed on a Hypersil Gold C18 column (100 mm × 2.1 mm i.d., 1.9 µm particle diameter, end-capped) maintained at 30 °C. The mobile phase consisted of (A) acetonitrile and (B) 0.1% formic acid (*v*/*v*). The gradient program started at 5% A and increased to 40% A over 14.72 min, followed by a ramp to 100% A over 1.91 min, held for 2.19 min, and then returned to initial conditions. The flow rate was set at 0.2 mL/min. UV–Vis spectral data were acquired in the range of 200–500 nm, and chromatograms were recorded at 280 and 340 nm. Data acquisition and control were performed using the Thermo Xcalibur Qual Browser Software (Thermo Scientific, San Jose, CA, USA). Nitrogen (purity >99%) was used as the nebulizing gas at a pressure of 520 kPa (75 psi). The mass spectrometer was operated in negative-ion mode, with an ESI needle voltage of 5.00 kV and a capillary temperature of 275 °C. Full-scan mass spectra were recorded over an *m*/*z* range of 100–2000. CID–MS/MS and MS^n^ experiments were performed using helium as the collision gas, with collision energies set at 25–35 arbitrary units.

### 2.2. Mice

Thirty female *Mus musculus* of the FVB/n strain were used to evaluate the rhizome extract: twenty transgenic (HPV+) and ten wild-type (WT/HPV−) mice aged 35–37 weeks. Another thirty female *Mus musculus* of the FVB/n strain were used to evaluate the effects of the fiddlehead extract: twenty transgenic (HPV+) and ten wild-type (WT/HPV−) mice aged 23–25 weeks. This age range was selected because K14-HPV16 mice typically develop dysplastic skin lesions between 12 and 24 weeks of age [20], allowing the assessment of potential carcinogenic co-factors during this period. Female mice were chosen to reduce aggressive behavior among animals, which is more common in male animals [21]. The mouse strain was generously provided by Drs. Jeffrey Arbeit and Douglas Hanahan from the University of California, through the National Cancer Institute Mouse Repository (USA) [16,20]. The animals were genotyped using a polymerase chain reaction (PCR) technique, as previously described [22].

### 2.3. Experimental Procedures

This experimental work was conducted at the animal facility of the University of Trás-os-Montes and Alto Douro (UTAD) with approval from the Ethics Committee (approval no. 852-e-CITAB-2020_A_1-e- 122 CITAB-2021) and the Portuguese Veterinary Directorate (approval no. 014139). The mice were housed in a laboratory animal facility under controlled environmental conditions, including temperature (21–25 °C), a 12 h light/dark cycle, and relative humidity (40–60%). The animals were fed a standard laboratory diet (4RF21 GLP, Mucedola, Italy) *ad libitum* and had free access to tap water according to the experimental design.

**Figure 1:**
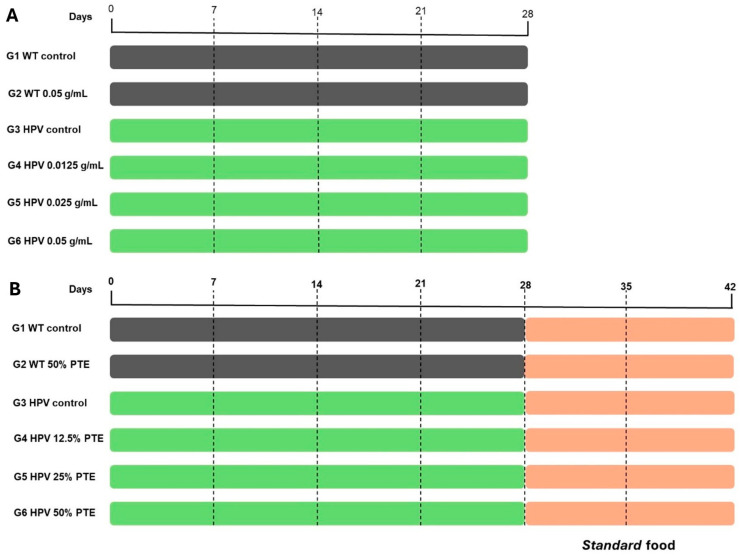
Experimental design. (**A**) Female mice were exposed daily to oral administration of an extract of *Pteridium aquilinum* rhizomes. Body weight, humane endpoints, and food and water consumption were recorded weekly. After 28 days, the animals were euthanized in accordance with FELASA recommendations. (**B**) Female mice were exposed daily to a diet containing freeze-dried *Pteridium aquilinum* fiddleheads. The animals received the supplemented diet for 28 days, followed by 15-day period on a standard diet. Body weight, humane endpoints, and food and water consumption were recorded weekly. After 42 days, the animals were euthanized in accordance with FELASA recommendations.

For the rhizome extract assay, mice were divided into six groups (n = 5): group 1 (G1, WT, control), group 2 (G2, WT, 0.050 g/mL), group 3 (G3, HPV, control), group 4 (G4, HPV, 0.0125 g/mL), group 5 (G5, HPV, 0.025 g/mL) and group 6 (G6, HPV, 0.050 g/mL) (Figure 1A). The extract of *Pteridium aquilinum* rhizomes was orally administered via drinking water for 28 days and was renewed every two days.

For the freeze-dried fiddlehead assay, mice were divided into six groups (n = 5): group 1 (G1, WT, control), group 2 (G2, WT, 50% *Pteridium aquilinum* (PTE) diet), group 3 (G3, HPV, control), group 4 (G4, HPV, 12,5% PTE diet), group 5 (G5, HPV, 25% PTE diet) and group 6 (G6, HPV, 50% PTE diet) (Figure 1B). The diet containing freeze-dried *Pteridium aquilinum* fiddleheads was administered for 28 days, followed by a 15-day period during which the animals were maintained on the standard diet.

Body weight, food intake, and water consumption were recorded every eight days. Humane endpoints were assessed weekly using a composite scoring system that evaluated parameters such as body weight, fur condition, eye and ear health, whisker appearance, mental alertness, and the presence of papilloma, among others, as detailed by Silva-Reis et al. (2021) [23]. A cumulative score was calculated, and animals reaching a predefined threshold of four were humanely euthanized prior to the end of the experiment [24]. At the end of this study, all animals were euthanized via intraperitoneal injection of a 10:1 ratio of ketamine (100 mg/kg) to xylazine (10 mg/kg), followed by exsanguination through cardiac puncture, in accordance with the guidelines established by the Federation of European Laboratory Animal Science Associations (FELASA) [25]. Blood and organs were subsequently collected for analysis.

#### 2.3.1. Sample Collection and Hematology and Biochemical Analyses

Blood samples were collected in the morning from fasted mice via cardiac puncture using a 27-gauge needle (Henke Sass Wolf GmbH, Tuttlingen, Germany) into 0.5 mL lithium heparin tubes (FL medical). Hematological parameters were determined using a ProCyte Dx Hematology Analyzer (IDEXX Laboratories, Inc., Westbrook, ME, USA) and included red blood cell count (RBC), hematocrit (HCT), hemoglobin concentration (HB), mean corpuscular volume (MCV), mean corpuscular hemoglobin (MCH), mean corpuscular hemoglobin concentration (MCHC), red cell distribution width (RDW), white blood cell count (WBC), and WBC differential total count and percentage of neutrophils (NEU), lymphocytes (LYM), monocytes (MONO), eosinophils (EOS) and basophils (BASO), as well as platelet count (PLT), mean platelet volume (MPV), platelet distribution width (PDW), and plateletcrit (PCT). For serum glucose determination, animals fasted for 12 h. After this period, blood glucose levels were measured using a GlucoMen areo 2K glucometer with GlucoMen areo Sensor strips (A. Menarini Diagnostics S.r.l., Firenze, Italy). Albumin (ACCENT–200; ref: 7–238), alanine aminotransferase (ACCENT–200; ref:7–216), creatinine (ACCENT–200; ref: 7–233) and urea (ACCENT–200; ref: 7–206) were analyzed using the CORMAY ACCENT MC240 automated analyzer.

#### 2.3.2. Histological Analysis

Formalin-fixed samples were paraffin-embedded and stained with hematoxylin and eosin (H&E) for routine histopathological analysis under light microscopy using an Axioplan 2 microscope (Zeiss, Jena, Germany). Image analysis was performed using the LAS Advanced Analysis Software Package (no.: 12730448). Skin samples (ear and chest) were classified as previously described [26], including sebaceous hyperplasia; orthokeratosis hyperkeratosis; parakeratosis hyperkeratosis; papilloma; hyperplasia; inflammatory infiltrate; dermoid cyst; dysplasia; ulcer; carcinoma in situ; squamous cell carcinoma; and invasive carcinoma. Liver and spleen samples were classified as previously described [27]. Liver alterations included extramedullary hematopoiesis, inflammatory infiltrate, tumefaction, mononuclear infiltrate, subacute multifocal hepatitis, and necrosis. Spleen alterations included lymphoid hyperplasia, hemosiderosis, and inflammatory infiltrate.

#### 2.3.3. Comet Assay

The alkaline comet assay (pH > 13) was performed on mononuclear blood cells using four slides *per* animal (two slides treated with formamidopyrimidine DNA glycosylase (FPG) and two without FPG), with each slide containing two gels. Four slides precoated with 1% normal melting point agarose were prepared for each mouse. Fifty microliters of whole mouse blood were collected and mixed with 600 µL of 1% low-melting-point agarose. Two drops (70 µL each) were placed on each precoated slide. The slides were then incubated in a lysis solution (2.5 M NaCl, 0.1 M EDTA, 10 mM Tris, 1% Triton X-100, pH 10) at 4 °C for 1 h and rinsed with a solution containing 40 mM HEPES, 0.1 M KCl, 0.5 mM EDTA, and 0.2 mg/mL bovine serum albumin (pH 8.0). FPG is used to specifically measure oxidative DNA damage by detecting oxidized purines and converting them into DNA single-strand breaks [28]. The enzyme was obtained from New England Biolabs^®^ (M0240S; 8000 U/mL) [29,30]. The slides, both with and without FPG treatment, were incubated in an alkaline electrophoresis solution (0.3 M NaOH and 1 mM EDTA) for 30 min at 4 °C, followed by electrophoresis for 30 min at 25 V and 300 mA. The cells were then neutralized with phosphate-buffered saline (PBS) and rinsed with distilled water. DNA was stained with 4,6-diamidino-2-phenylindole (DAPI) and visualized using a fluorescence microscope (OLYMPUS-R XC10, U-RFL-T, Hamburg, Germany). For visual assessment, comets were classified based on tail intensity (class 0—no damage; class 4—high damage) [30]. The total score, representing the genetic damage indicator (GDI), was calculated according to the following formula:

GDI = [(% nucleoids in class 0) × 0] + [(% nucleoids in class 1) × 1] + [(% nucleoids in class 2) × 2] + [(% nucleoids in class 3) × 3] + [(% nucleoids in class 4) × 4].

One hundred comets *per* gel were scored to obtain a GDI value ranging from 0 to 400 arbitrary units. The scores obtained after FPG treatment (GDI_FPG) were subtracted from the untreated GDI to quantify net enzyme-sensitive sites (NSS_FPG).

#### 2.3.4. Hepatic and Kidney Oxidative Stress

Samples of liver and kidney were collected and stored at −80 °C until analysis. The samples were cut into portions of approximately 0.1 mg and homogenized in a cold buffer solution (0.32 mM sucrose, 20 mM HEPES, 1 mM MgCl_2_ and 0.5 mM phenylmethylsulfonyl fluoride, pH 7.4). Total protein content was quantified using a microplate reader (Biotek MicroPlate Reader^®^) for the analysis of carbonyls (CARB), reactive oxygen species (ROS) and lipid peroxidation (LPO). Prior to quantification, samples were centrifuged at 15,000× *g* for 20 min at 4 °C. Protein quantification was then repeated to assess the activity of antioxidant enzymes, including superoxide dismutase (SOD), catalase (CAT), reduced glutathione (GSH), oxidized glutathione (GSSG), glutathione peroxidase (GPx), glutathione S-Transferase (GST). The supernatants were collected and analyzed to evaluate oxidative stress and antioxidant response. SOD activity was determined at 560 nm, whereas CAT activity was measured at 240 nm, as previously described [31]. The oxidative stress index (OSI) was calculated as the ratio between GSH and GSSG. Levels of GSH and GSSG were measured at 320 nm and 420 nm using derivatization with ortho-phthalaldehyde [32]. Reactive oxygen species (ROS) production was estimated using the 2,7-dichlorofluorescein diacetate (DCFDA) probe, with excitation at 485 nm and emission at 530 nm, as previously reported [33,34]. Malondealdehyde (MDA), an indicator of lipid peroxidation, was quantified using the thiobarbituric acid (TBA) method at 530 nm [34]. GPx activity was evaluated at 340 nm by monitoring NADPH oxidation [35]. GST activity was measured based on the reaction between glutathione and 1-chloro-2,4-dinitrobenzene (CDNB) at 340 nm, using a molar extinction coefficient of 9.60 mM^−1^ cm^−1^. All analyses were performed using either a PowerWave XS2 microplate scanning spectrophotometer (BioTek Instruments, Santa Clara, CA, USA) or a Varian Cary Eclipse Spectrofluorometer (Varian; Palo Alto, CA, USA) equipped with a microplate reader.

### 2.4. Statistical Analysis

IBM SPSS Statistics for Windows, version 26 (IBM Corp., Armonk, NY, USA), and GraphPad Prism version 9 were used. Normality was assessed using the Shapiro–Wilk test, and homogeneity of variances was evaluated using the Levene test. Data following a normal distribution and homogeneity of variances were analyzed using one-way analysis of variance (ANOVA), followed by the Bonferroni post hoc test for multiple comparisons.

For data that did not meet parametric assumptions, non-parametric analyses were performed using the Kruskal–Wallis test, followed by appropriate multiple-comparison procedures when applicable. Statistical significance was defined as *p* < 0.05. The parameters analyzed using these approaches included body weight, food and water consumption, organ mass, humane endpoints, hematological variables, and oxidative stress parameters.

The association between histological lesions and experimental groups was assessed using the Chi-square test, as these data are categorical and expressed as frequencies.

The following formulas were used to calculate humane endpoint scores and corresponding weights:

▪ Humane endpoints = (sum score *per* animal)/(number of animals *per* cage).
▪ Weight gain = ((final weight − initial weight)/(initial weight)) × 100 (%).
▪ Average daily consumption (drink/food) *per* animal *per* day = ((initial weight) − (final weight))/(number of days between weightings) × (number of animals *per* cage) (g).
▪ Relative weight of organs = (organ weight)/(body weight) (mg/g).

## 3. Results

### 3.1. Chemical Composition

The UHPLC-ESI-MS analysis revealed clear differences in the chemical profiles of *Pteridium aquilinum* rhizomes and fiddleheads. The rhizome extract was predominantly characterized by pteroside derivatives, which were identified based on their typical UV spectra and MS/MS fragmentation patterns [36]. These compounds were the major constituents detected and are of particular toxicological interest given their structural relationship with known bioactive illudane-type compounds [36,37,38].

In addition to pterosides, rhizomes also contained a limited number of phenolic compounds, including caffeic acid derivatives and flavonoid-related structures, as well as dihydrobenzofuran neolignanes such as brainic acid and blechnic acid derivatives [39,40,41,42,43].

In contrast, the chemical composition of fiddleheads was markedly different, being dominated by phenolic compounds. These included caffeoyl-shikimate isomers, tryptophan–phenolic conjugates, and flavonoids, mainly kaempferol derivatives. This profile suggests a greater prevalence of antioxidant-related compounds in fiddleheads compared to rhizomes [39].

Importantly, no ptaquiloside or related illudane-type glycosides were detected in the fiddlehead samples under the experimental conditions. This absence may be explained by the extraction procedure, including washing steps and solvent selection, which may have reduced or eliminated these highly water-soluble compounds [43,44,45,46,47,48,49,50,51].

**Figure 2:**
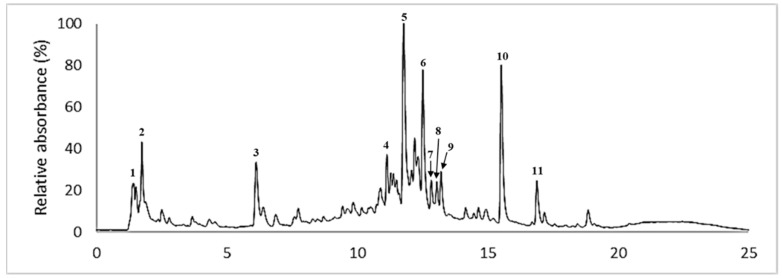
Chromatographic profile of *Pteridium aquilinum* rhizome extract recorded at 280 nm. Numbers on top of each peak correspond to the UHPLC-ESI-MS_n_ peaks listed in Table 1 and Supplementary Materials.

**Figure 3:**
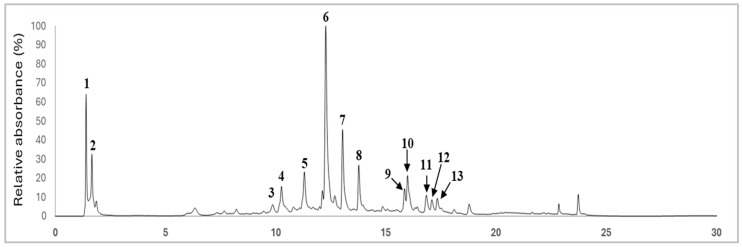
Chromatographic profile of freeze-dried fiddleheads of *Pteridum aquilinum.,* recorded at 280 nm. Numbers on top of each peak correspond to the UHPLC-ESI-MS_n_ peaks listed in Table 2.

Detailed MS/MS fragmentation data supporting compound identification are provided in Figure 2 and Figure 3 and Table 1 and Table 2.

### 3.2. General Findings

There were no observable phenotypic or behavioral changes in the experimental groups in both assays. Furthermore, no mortality or early euthanasia events were recorded during the experimental period, and no animal reached the predefined humane endpoint threshold. Nevertheless, variations in humane endpoint scores were observed among groups, with HPV16 transgenic mice exposed to rhizome extracts showing a trend toward higher scores compared to controls (Table 3). The scoring system integrates multiple indicators of animal welfare, including body condition, behavior, and clinical signs, allowing early detection of physiological impairment even in the absence of severe endpoints.

Regarding food and drink consumption, HPV16 animals showed higher values compared to WT animals when exposed to the rhizome extract. In the case of freeze-dried fiddleheads, food consumption was similar between groups, while water consumption was higher in HPV16 animals compared to WT animals (Table 3). It should be noted that exposure levels are presented as administered concentrations, as actual intake depends on individual food and water consumption. Therefore, concentration estimates should be considered approximate rather than absolute.

In general, in the rhizome assay, WT mice showed higher body weight compared to HPV16 mice. While WT animals exposed to the extract exhibited weight gain, HPV16 animals showed reduced weight gain. Statistically significant differences (*p* < 0.05) were observed between G2 (WT, 0.05 g/mL) and G6 (HPV, 0.05 g/mL) (Table 3). In the fiddlehead assay, the opposite pattern was observed: WT mice exposed to the extract had lower average body weight compared to their control group, whereas HPV16 mice showed higher body weight compared to their respective controls. However, there were no statistical differences (*p* > 0.05).

In the rhizome assay, the mean relative heart weight was consistent across all groups. For lung weight, an increasing trend was observed in HPV groups with higher extract concentrations (Table 4). HPV groups, particularly G3 (HPV, control) and G6 (HPV, 0.05 g/mL), showed higher mean relative spleen weight compared to WT (G1 and G2). This increase was more evident at higher extract concentrations (0.025 and 0.05 g/mL). The mean relative liver weight was consistently higher in the HPV groups compared to the WT groups, with a notable increase at higher extract concentration. Statistically significant differences were observed between G2 (WT, 0.05 g/mL) and G6 (HPV, 0.05 g/mL), as well as between G3 (HPV, control) and G6 (HPV, 0.05 g/mL) (*p* < 0.05). Regarding kidney weight, G3 (HPV, control) showed the highest mean relative weight among groups, although no clear trend with increasing extract concentration was observed in HPV mice. In the fiddlehead assay, no clear trends were observed for mean relative heart weight. Mean relative lung weight was higher in G3 (HPV, control) compared to G1 (WT, control) and showed a slight decrease with increasing PTE concentration in WT mice (G2) and a slight increase in HPV mice (G6). HPV groups (G3, G5 and G6) generally showed higher mean relative spleen weight compared to WT groups (G1 and G2). A reduction in spleen weight was observed in G2 (WT, 50% PTE) compared to G1 (WT, control), like the trend in HPV groups. Mean relative liver weight was significantly higher in HPV groups compared to WT groups, except for G6 (HPV, 50% PTE). Although an increase was observed in G6 (HPV, 50% PTE) compared to G3 (HPV, control), this trend was not consistent across intermediate concentrations. A statistically significant difference was found between G1 (WT, control) and G3 (HPV, control) (*p* < 0.05). Regarding kidney weight, G3 (HPV, control) showed the highest value, and no consistent trend with increasing PTE concentration was observed in HPV groups. Statistically significant differences were observed between groups G1 (WT, control) and G3 (HPV, control), and between G2 (WT, 50% PTE) and G6 (HPV, 50% PTE) (*p* < 0.05) (Table 4).

### 3.3. Hematology and Biochemical Analyses

Table 5 presents the hematological results for rhizome extract. Regarding hematocrit, hemoglobin, lymphocytes, erythrocyte values, and G3 (HPV, control) showed reduced levels compared to the other groups. Neutrophils and leukocytes showed higher mean values in G6 (HPV, 0.05 g/mL) compared to the other groups, particularly when compared to the control group G3 (HPV, control). Monocytes, eosinophils, and basophils did not show significant variations between groups. Platelet counts were higher in G4 (HPV, 0.0125 g/mL) and G5 (HPV, 0.025 g/mL), while reticulocyte values were lower in G6 (HPV, 0.05 g/mL). MPV, PDW, MCV, MCH, and MCHC indices showed limited variation between groups, with some significant exceptions. G6 (HPV, 0.05 g/mL) exhibited higher MCV values, indicating larger red blood cells. Mean glucose levels are lower in G4 (HPV, 0.0125 g/mL), G5 (HPV, 0.025 g/mL) and G6 (HPV, 0.05 g/mL). Total protein and albumin levels were higher in G2 (WT, 0.05 g/mL), G4 (HPV, 0.0125 g/mL), G5 (HPV, 0.025 g/mL), and G6 (HPV, 0.05 g/mL). Alanine aminotransferase (ALAT) showed higher mean values in G3 (HPV, control) and G5 (HPV, 0.025 g/mL).

Table 6 presents the hematological results of the fiddlehead assay. Regarding hematocrit and hemoglobin, HPV16 groups (G3 (HPV, control); G4 (HPV, 12.5% PTE); G5 (HPV, 25% PTE) and G6 (HPV, 50% PTE)) showed lower values compared to WT groups (G1 (WT, control) and G2 (WT, 50% PTE)). In HPV16 groups, a slight decrease in neutrophils and an increase in lymphocytes were observed. Monocyte and eosinophil levels decreased in G4 (HPV, 12.5% PTE), G5 (HPV, 25% PTE), and G6 (HPV, 50% PTE) compared to G3 (HPV, control). Basophil levels remained relatively uniform across all groups, with no significant differences. Platelet counts were significantly higher in G4 (HPV, 12.5% PTE) and G5 (HPV, 25% PTE) compared to G3 (HPV, control). Erythrocyte values were consistent across all groups, with no significant differences. Reticulocyte counts decreased following administration of the freeze-dried *Pteridium aquilinum* fiddleheads. MPV, PDW, MCV, MCH, and MCHC indices showed minimal variation between groups, with no statistically significant differences. Glucose levels were higher in G3 (HPV, control) compared to the groups treated with freeze-dried fiddleheads. Total protein levels showed a slight decrease in HPV16 groups, with no major differences between groups treatment concentrations. Albumin levels were consistently lower in HPV16 groups, except for G5 (HPV, 25% PTE). ALAT presents higher average values in G3 (HPV, control), while treated groups exhibited a slight reduction.

### 3.4. Comet Assay

**Figure 4:**
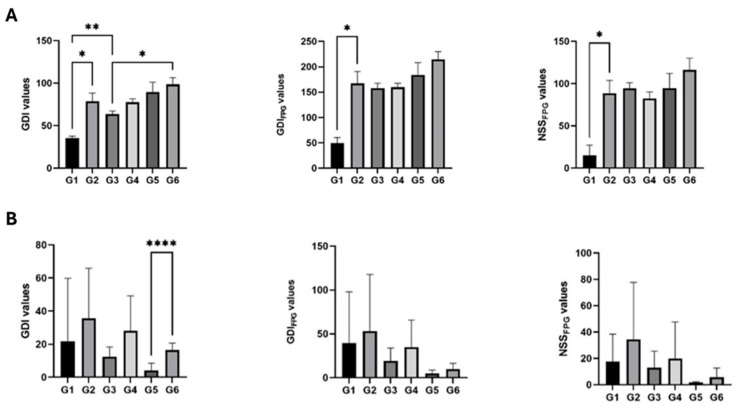
Genetic damage index (GDI) values determined by the comet assay (mean ± standard deviation; expressed as arbitrary units): (**A**) Assay 1: infusion of aqueous extract of Pteridium aquilinum rhizomes. (**B**) Assay 2: freeze-dried Pteridium aquilinum fiddlehead extract. Parameters: GDI—basal damage, GDI_FPG—total damage and NSS_FPG—oxidative damage. Groups: G1 (WT, control), G2 (WT, 50% PTE), G3 (HPV, control), G4 (HPV, 12.5% PTE), G5 (HPV, 25% PTE), G6 (HPV, 50% PTE). Statistical significance was defined as *p* ≤ 0.05 (*), *p* ≤ 0.01 (**) and *p* ≤ 0.0001 (****).

In the rhizome assay, GDI values for G2 (WT, 0.05 g/mL) and G3 (HPV, control) were significantly higher than those of G1 (WT, control). In addition, G6 (HPV, 0.05 g/mL) showed significantly higher values than G3 (HPV, control). Regarding GDI_FPG and NSS_FPG, G2 (WT, 0.025 g/mL) exhibited significantly higher values compared to G1 (WT, control) (*p* < 0.05) (Figure 4A). In the fiddlehead assay, GDI values were higher in G6 (HPV, 50% PTE) compared to G5 (HPV, 25% PTE) (*p* < 0.05) (Figure 4B).

### 3.5. Histology

**Figure 5:**
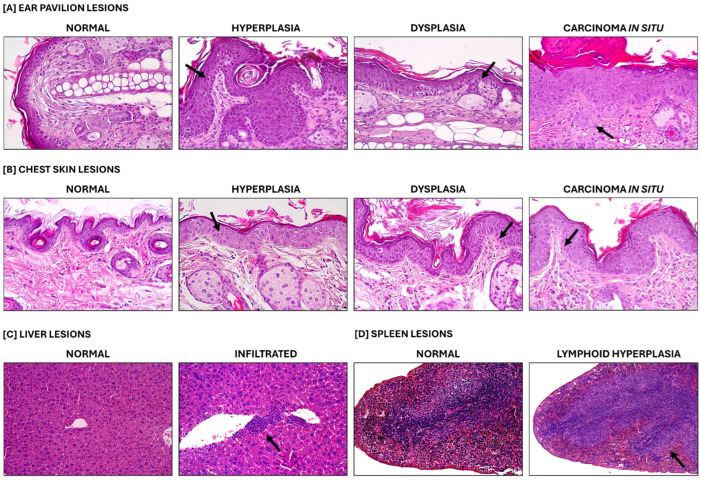
Histopathological features observed in wild-type (WT) and HPV16 transgenic mice. Hematoxylin and eosin stain. (**A**) Ear pavilion lesions: normal skin histology (WT), 400×. Hyperplasia, 400×. Dysplasia, 400×. Carcinoma in situ, 400×. (**B**) Chest skin lesions: Normal skin histology (WT), 400×. Hyperplasia, 400×. Dysplasia, 400×. Carcinoma in situ, 400×. (**C**) Liver: normal (WT), 200×. Inflammatory infiltrate, 200×. (**D**) Spleen: normal (WT), 200×. Hyperplasia, 100×.

Histopathological evaluation revealed a spectrum of lesions reflecting progressive epithelial alterations. Dysplasia was characterized by abnormal cellular organization and nuclear atypia, while carcinoma in situ involved full-thickness epithelial changes without evidence of invasion. Inflammatory infiltrates were also frequently observed, indicating an ongoing inflammatory response. The histological results of the rhizome assay are presented in Table 7 and Figure 5. As expected, cutaneous lesions were observed exclusively in the HPV groups. In the ear pavilion skin, the orthokeratotic hyperkeratosis increased with extract concentration (G4 (HPV, 0.0125 g/mL), G5 (HPV, 0.025 g/mL), G6 (HPV, 0.05/mL)). Parakeratotic hyperkeratosis was observed in all animals in G3 (HPV, control), with statistically significant differences between G1 (WT, control) and G3 (HPV, control) (*p* < 0.05), G2 (WT, 0.05 g/mL) and G6 (HPV, 0.05 g/mL) (*p* < 0.05), and G3 (HPV, control) and G5 (HPV, 0.025 g/mL) (*p* < 0.05). Papilloma, hyperplasia, dysplasia, ulcers, carcinoma in situ, squamous cell carcinoma, and invasive carcinoma were observed across multiple treated groups, with a higher frequency at increasing extract concentrations (G4 (HPV, 0.0125 g/mL), G5 (HPV, 0.025 g/mL), G6 (HPV, 0.05/mL). Statistically significant differences were observed in hyperplasia and dysplasia parameters between G1 (WT, control) and G3 (HPV, control), as well as between G2 (WT, 0.05 g/mL) and G6 (HPV, 0.05 g/mL) (*p* < 0.05). Chest skin lesions showed a similar trend to those observed in the ear pavilion, with increased incidence of orthokeratotic and parakeratotic hyperkeratosis at higher concentrations of the aqueous extract from *Pteridium aquilinum* rhizomes. Orthokeratotic hyperkeratosis showed statistically significant differences between G2 (WT, 0.05 g/mL) and G6 (HPV, 0.05 g/mL), as well as between G5 (HPV, 0.025 g/mL) and G6 (HPV, 0.05 g/mL) (*p* < 0.05). Parakeratotic hyperkeratosis also exhibited statistically significant differences between G4 (HPV, 0.0125 g/mL) and G6 (HPV, 0.05 g/mL), and between G5 (HPV, 0.025 g/mL) and G6 (HPV, 0.05 g/mL) (*p* < 0.05). Papilloma, hyperplasia, dysplasia, ulcers, carcinoma in situ, squamous cell carcinoma, and invasive carcinoma were observed more frequently in groups exposed to higher concentrations (G4 (HPV, 0.0125 g/mL), G5 (HPV, 0.025 g/mL), G6 (HPV, 0.05/mL)). In the liver (Figure 5C), extramedullary hematopoiesis was observed at low incidence with no clear concentration-dependent trend. In G2 (WT, 0.05 g/mL), mononuclear infiltrate levels were significantly higher than in G6 (HPV, 0.05 g/mL) (*p* < 0.05). Subacute multifocal hepatitis and necrosis were observed in some animals from the treated groups, particularly at the highest concentrations (G5 (HPV, 0.025 g/mL), G6 (HPV, 0.05/mL). In the spleen (Figure 5D), lymphoid hyperplasia and hemosiderosis lesions were more frequent in G2 (WT, 0.05 g/mL), as well as in G5 (HPV, 0.025 g/mL) and G6 (HPV, 0.05 g/mL).

The histological results of the fiddlehead assay are presented in Table 8 and Figure 5. As expected, cutaneous lesions were observed exclusively in HPV groups, while all animals in the WT groups showed normal (0%) auricular and chest skin, indicating that the extract did not induce damage in these tissues. Lesions were observed in all HPV groups. In ear pavilion lesions, sebaceous hyperplasia showed 100% prevalence in G6 (HPV, 50% PTE). Statistically significant differences were observed between G2 (WT, 50% PTE) and G6 (HPV, 50% PTE), G3 (HPV, control) and G6 (HPV, 50% PTE), and G4 (HPV, 12.5% PTE) and G6 (HPV, 50% PTE) (*p* < 0.05). Hyperkeratosis, parakeratotic changes, and inflammatory infiltrate were increased in G4 (HPV, 12.5% PTE), G5 (HPV, 25% PTE), and G6 (HPV, 50% PTE). In chest skin, sebaceous hyperplasia was the predominant lesion in G6 (HPV, 50% PTE), while inflammatory infiltrates were more frequent in G3 (HPV, control), G4 (HPV, 12.5% PTE), and G6 (HPV, 50% PTE). In the liver (Figure 5C), inflammatory infiltrate was more pronounced in G2 (WT, 50% PTE), G5 (HPV, 25% PTE), and G6 (HPV, 50% PTE), with tumefaction observed in G4 (HPV, 12.5% PTE), G5 (HPV, 25% PTE), and G6 (HPV, 50% PTE). In the spleen (Figure 5D), inflammatory infiltrates were particularly evident in G4 (HPV, 12.5% PTE) and G5 (HPV, 25% PTE).

### 3.6. Hepatic and Renal Oxidative Stress

**Figure 6:**
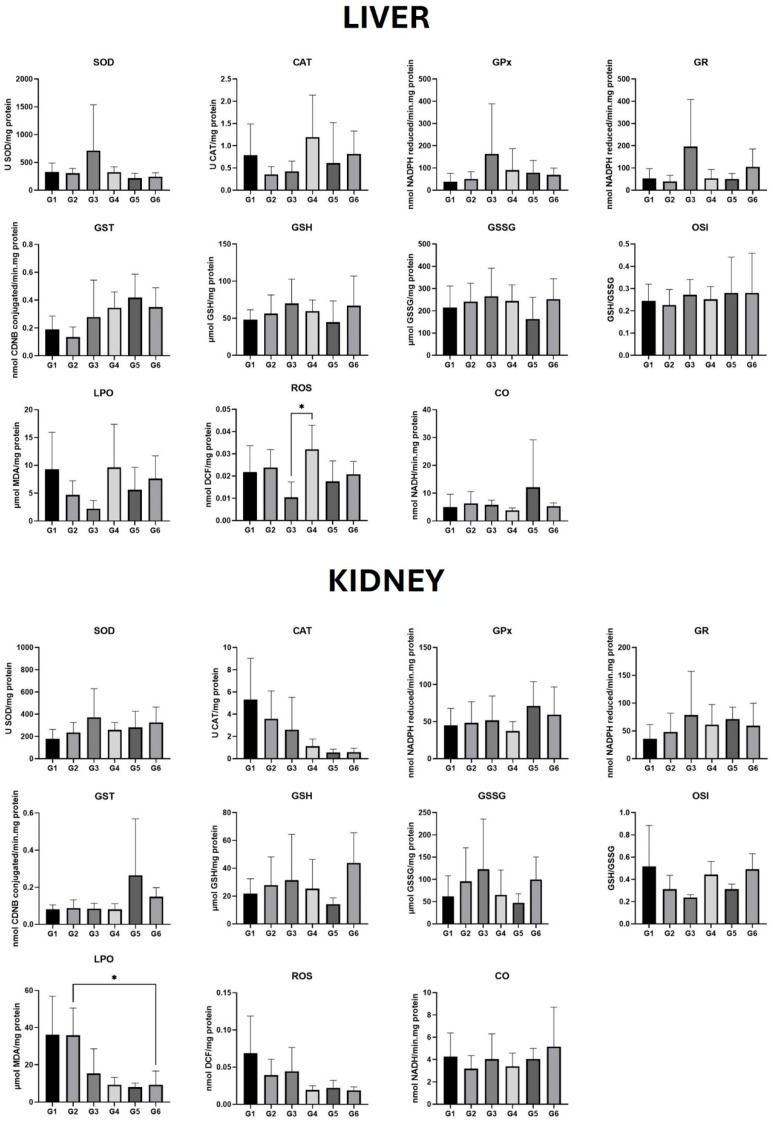
Liver and kidney oxidative stress parameters of the assay from aqueous extract from *Pteridium aquilinum* rhizomes. Cu/Zn-SOD (U SOD/mg protein); CAT (U CAT/mg protein); GPx (nmol NADPH reduced/min·mg protein); GR (nmol NADPH reduced/min·mg protein); GST (nmol CDNB conjugated/min·mg protein); GSH (µmol GSH/mg protein); GSSG (µmol GSSG/mg protein); OSI (GSH/GSSG); LPO (µmol MDA/mg protein); ROS (nmol DCF/mg protein); and CO (nmol NADH/min·mg protein). In liver, statistically significant differences in the ROS levels were observed between G3 (HPV, control) and G4 (HPV, 0.025 g/mL) (*p* < 0.05). In kidney, statistically significant differences in the LPO levels between G2 (WT, 0.05 g/mL) and G6 (HPV, 0.05 g/mL) (*p* < 0.05). * indicates statistically significant differences (*p* ≤ 0.05).

**Figure 7:**
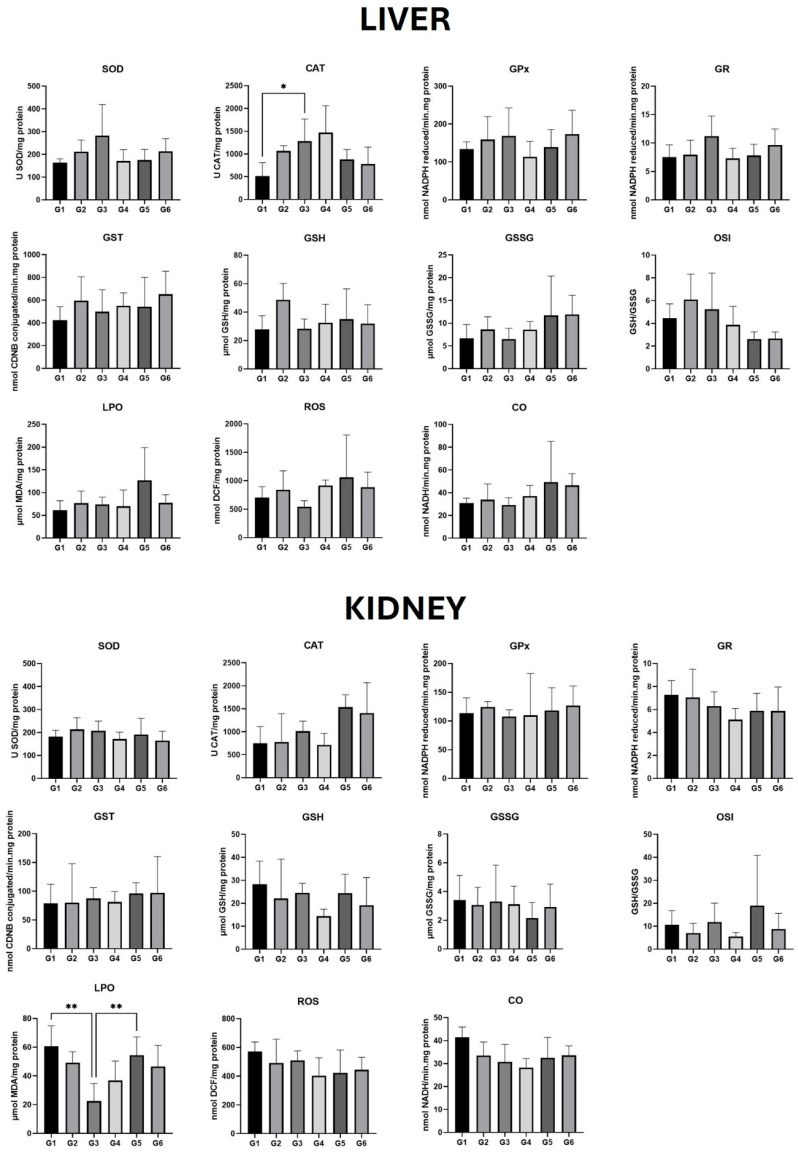
Liver and kidney oxidative stress paramerts of the assay from freeze-dried fiddleheads of *Pteridium aquilinum* Cu/Zn-SOD (U SOD/mg protein); CAT (U CAT/mg protein); GPx (nmol NADPH reduced/min·mg protein); GR (nmol NADPH reduced/min·mg protein); GST (nmol CDNB conjugated/min·mg protein); GSH (µmol GSH/mg protein); GSSG (µmol GSSG/mg protein); OSI (GSH/GSSG); LPO (µmol MDA/mg protein); ROS (nmol DCF/mg protein); and CO (nmol NADH/min·mg protein). In liver, statistically significant differences in the CAT levels between G1 (WT, control) and G3 (HPV, control) (*p* < 0.05). In kidney, statistically significant differences in the LPO levels between G1 (WT, control) and G3 (HPV, control) (*p* < 0.05), and G3 (HPV, control) and G5 (HPV, 25% PTE) (*p* < 0.05). Statistical significance was defined as *p* ≤ 0.05 (*), *p* ≤ 0.01 (**).

In the rhizome assay, oxidative stress analysis in the liver and kidney (Figure 6) revealed significant differences in ROS levels in the liver and LPO in the kidney. Liver ROS levels were significantly higher in G4 (HPV, 0.025 g/mL) compared to G3 (HPV, control) (*p* < 0.05) (Figure 6—liver). In the kidney, the only significant difference observed was a decrease in LPO levels in G6 (HPV, 0.05 g/mL) compared to G2 (WT, 0.05 g/mL) (*p* < 0.05) (Figure 6—kidney). In the fiddlehead assay, oxidative stress analysis (Figure 7) showed significant differences in CAT activity in the liver and LPO levels in the kidney. Liver CAT activity was significantly higher in G3 (HPV, control) compared to G1 (WT, control) (*p* < 0.05) (Figure 7—liver). In the kidney, LPO levels were significantly lower in the G3 (HPV, control) compared to G1 (WT, control) and G5 (HPV, 25% PTE) (*p* < 0.05) (Figure 7—kidney).

## 4. Discussion

The bracken fern (*Pteridium aquilinum*) is a highly problematic plant worldwide due to its toxicity combined with its invasive properties in formerly agricultural lands, deforested areas, and disturbed natural habitats [7,51]. The carcinogenic potential of bracken ferns has been a cause of scientific and public concern for six decades [51]. Their genotoxic effects are associated with iludane-type glycosides (ITGs), their aglycones, and derivatives [4]. Ptaquiloside is generally considered the most common ITG, but other ITGs also exist, and their contributions remain largely understudied [52]. A diet that includes *Pteridium* spp. can lead to various symptoms in animals and humans [53]. These include neurological disorders linked to thiamine deficiency, particularly seen in horses; acute hemorrhagic disease in cattle and sheep; retinal atrophy in sheep; and enzootic hematuria in cattle, which is associated with the development of cancer in the bladder and digestive tract [54]. In humans, there is limited evidence associating exposure to *Pteridium* spp. and digestive tract cancers [17]. The ingestion of toxins found in *Pteridium* spp. facilitates PV infection in animals and humans, particularly those in the field, promoting the development of cancer linked to this agent [55]. Interestingly, a previous study by our group using K14-HPV16 mice showed that ptaquiloside from bracken fern was also able to promote oral carcinogenesis, which is initiated by HPV16 in this model [56,57]. Taking this into consideration, this study addressed the in vivo effects of an aqueous extract of *Pteridium* spp. rhizomes and freeze-dried fiddleheads of *Pteridium* spp., in K14-HPV16 mice. Chemical analysis did not reveal ITGs in any of the products administered. Therefore, the effects observed in this animal model under these experimental conditions may be attributed to other chemical compounds present. It is worth noting that compound Blechinic acid has not been previously reported in this plant.

In both assays, all mice survived the experimental protocol, with no observable changes in the animals’ behavior. The concentrations tested in both studies did not adversely affect animal welfare, as indicated by the humane endpoint scoring system. Regarding body weight and weight gain, the results suggest that rhizome extracts may have differential effects depending on the animal model. Specifically, WT mice showed increased weight gain following exposure, whereas HPV16 transgenic mice exhibited reduced weight gain. They may reflect differences in physiological response between healthy and transgenic mice. It is possible that bioactive compounds in *Pteridium aquilinum*, such as pteroside derivatives (e.g., pteroside D and X/W), may contribute to immunomodulatory effects [58], as previously suggested by Mohammad and collaborators in 2016. Moreover, the reduced weight gain observed in HPV16 mice may be related to the characteristics of this model, which is often associated with features of cachexia. Cachexia is a multifactorial syndrome characterized by systemic inflammation, weight loss and muscle loss [59], and this condition may be exacerbated in HPV16 transgenic animals under certain experimental conditions. In contrast, in the fiddlehead assay, all animals exhibited increased body weight and weight gain. These findings suggest that exposure to freeze-dried fiddleheads was associated with more stable growth patterns in both WT and HPV16 mice under the conditions tested.

Food and water consumption were higher in HV1P16 transgenic mice. These results are consistent with previous findings from our group [57,60], suggesting that HPV-induced epidermal alterations may lead to dehydration and increased water intake [61,62,63,64]. Among rhizome-exposed animals, the liver was the only organ showing a statistically significant increase in relative weight in G6 (HPV, 0.05 g/mL) compared to G2 (WT, 0.05 g/mL) and G3 (HPV, control). In the fiddlehead assay, increased liver weight was also observed in G3 (HPV, control) compared to G1 (WT, control), while kidney weight was higher in G6 (HPV, 50% PTE) compared to G1 (WT, control) and G2 (WT, 50% PTE). It is known that HPV16 transgenic mice tend to exhibit increased liver mass due to chronic inflammation associated with transgene expression [26]. In the present study, exposure to rhizome extract appeared to further enhance this effect, although no clear concentration-dependent pattern was observed [63].

Chronic consumption of *Pteridium aquilinum* has been associated with hematological alterations, including anemia, leukopenia, monocytosis, thrombocytopenia, hypergammaglobulinemia, microhematuria, and proteinuria [65]. Monocytosis, in particular, has been suggested as an early marker of exposure [65]. However, the hematological findings in the present study differed from those reported in previous studies. Mice exposed to rhizome extract showed increased hematocrit values, which may reflect dehydration [66,67], enhanced erythropoiesis, or age-related changes [68]. Lymphocyte levels decreased in WT mice following supplementation, while leukocyte counts were not markedly affected [62]. Platelet counts increased in both WT and HPV16 mice. Additionally, glucose levels increased in WT mice exposed to rhizomes but decreased in HPV16 mice, suggesting differential metabolic responses. Similar results were observed in a trial on Wistar rats, in which a formulation with *Pteridium aquilinum*, *Mucuna puriens* and *Newbouldia laevis* incorporated into the animals’ diets allowed the reduction in glucose in the blood of these animals [69]. In contrast, no significant hematological alterations were observed in the fiddlehead assay. These differences may reflect variations in chemical composition, exposure route, and bioavailability between treatments.

Both rhizome extract and freeze-dried fiddleheads were associated with increased DNA damage in peripheral blood cells, as indicated by the alkaline comet assay. There is substantial scientific evidence from the assessment of the genotoxicity of ITGs and *Pteridium aquilinum* that they have harmful effects [70]. Although ITGs were not detected, these findings suggest that other compounds present in *Pteridium aquilinum* may contribute to genotoxic effects. Also, although fiddleheads are considered the most toxic part of bracken fern, rhizomes should also be considered with care, as they also show genotoxic potential.

The greater toxicological impact observed for rhizome extracts, compared with fiddleheads, is noteworthy, particularly considering that fiddleheads are often reported to contain higher levels of ptaquiloside. This apparent discrepancy may be explained by several factors. First, differences in sample processing and extraction procedures may have influenced the stability and recovery of labile compounds such as ptaquiloside. Second, rhizomes may contain additional bioactive or synergistic metabolites, including pteroside derivatives, which could contribute to the observed biological effects. Furthermore, differences in the route of exposure may have influenced compound bioavailability, as rhizome extracts were administered through drinking water, whereas fiddleheads were incorporated into the diet. These factors may have affected absorption, metabolism, and overall systemic exposure, thereby contributing to the distinct toxicological profiles observed between the two preparations.

It should also be considered that the different routes of exposure used in this study may have influenced compound intake and bioavailability. While rhizome extracts were administered through drinking water, fiddleheads were incorporated into the diet, which may have affected palatability, absorption, and overall systemic exposure. Although food and water consumption should be taken into account when directly comparing the toxicological effects of the two treatments.

Regarding the histological analyses of tissue samples following exposure to the aqueous extract of *Pteridium aquilinum* rhizomes, we observed significant pro-inflammatory and pro-carcinogenic effects in HPV-transgenic mice, and these effects appeared to be concentration-dependent. These findings support previous observational and experimental evidence, pointing to a systemic impact of *Pteridium aquilinum* on immune function. In a study conducted by Latorre and collaborators in 2008, the administration of *Pteridium aquilinum* by gavage in C57BL/6 mice at a dose of 30.0 g/Kg was found to cause a reduction of T and B lymphocyte counts. Consequently, the reduction in lymphocyte numbers may contribute to the carcinogenic effects induced by this plant [71]. Immune cell infiltration is a key factor in modulating carcinogenesis and contributes to several hallmarks of cancer [72]. Concerning the freeze-dried fiddleheads, a trend towards more pronounced inflammatory changes was observed. Overall, the histological findings underscore the potentially harmful effects of *Pteridium aquilinum*, particularly in the context of HPV infection, and highlight the importance of considering concentration-dependent effects when assessing its safety profile.

The analysis of oxidative stress revealed statistically significant hepatic differences in the ROS parameter that were associated with oral administration of the rhizome extract. In addition, statistically significant differences in the kidney were also observed in the LPO parameter, which were associated with the transgenesis of the mice. An antioxidant activity assay has been described in the literature, reporting that *Pteridium aquilinum* young fronds at concentrations of 5, 50 and 100 µg/mL exhibited good antioxidant activity, as demonstrated by the diphenyl-1-picrylhydrazyl radical (DPPH) technique [73]. In vitro antioxidant tests showed that the purified polysaccharide of *Pteridium aquilinum* possessed a strong free radical scavenging effect against superoxide radical, and exhibited inhibition of the auto-oxidation of 1,2,3-fentriol [74]. In the fiddlehead assay, it is noteworthy that the CAT parameter in the liver was statistically higher in G3 (HPV, control) compared to G1 (WT, control). This indicates possible metabolic changes caused by HPV that can lead to oxidative stress [75]. Consequently, this group may reflect a response to increased oxidative stress or other changes induced by viral infection, with enhanced CAT activity representing an adaptive mechanism to protect against oxidative damage. In the kidney, the LPO parameter is statistically higher in G5 (HPV, 25% PTE) compared to G3 (HPV, control). Additionally, G3 (HPV, control) is statistically lower than G1 (WT, control). This may be due to a concentration-dependent effect and tolerance limits. Wild-type mice can tolerate higher concentrations of Pteridium spp. well, benefiting from its antioxidant effects without reaching a toxicity threshold. However, they have a reduced tolerance to potentially toxic compounds in *Pteridium aquilinum*, which can act as pro-oxidants or become toxic under certain conditions.

In conclusion, the fiddleheads of *Pteridium aquilinum* were better tolerated than the rhizome extract of *Pteridium aquilinum,* as the aqueous rhizome extract affected body weight. Regarding histological lesions in the rhizome extract, the severity and prevalence of lesions increased with higher concentrations of *Pteridium aquilinum*, especially in HPV mice. This suggests that the compounds present in the extract mostly have a significantly negative impact when administered in high concentrations, even though ITGs were not detected. This was also in line with the induction of genotoxic damage in peripheral blood lymphocytes. In both assays, the mice with HPV showed greater susceptibility to the adverse effects of *Pteridium aquilinum*, with a significant increase in inflammatory and carcinogenic lesions. This may be due to a compromised antioxidant system or an exacerbated inflammatory response, highlighting the importance of considering the body’s health status when evaluating the risks of *Pteridium aquilinum* exposure. In summary, the results indicate that administering different parts of the *Pteridium aquilinum* plant to animals leads to varying outcomes. The extracts from the rhizomes were associated with more pronounced biological effects compared to fiddleheads, particularly in HPV. These damages are concentration-dependent and include inflammatory and carcinogenic lesions in the skin, liver, and spleen. Therefore, it is essential to consider these risks before consuming this plant, along with the preparation and cooking methods used.

Some limitations of this study should be acknowledged. The rhizome and fiddlehead assays differed in several experimental aspects, including route of administration (drinking water vs. dietary incorporation), exposure duration, the presence of a recovery period, and the age of the animals at the start of the experiments. These factors may influence compound intake, bioavailability, and biological responses, particularly in the K14-HPV16 model, where lesion development is age-dependent. Therefore, the two assays should be interpreted as parallel experimental approaches, and direct quantitative comparisons between them should be made with caution.

## 5. Conclusions

The results indicate that different parts of *Pteridium aquilinum* may elicit distinct biological responses under the present experimental conditions, with rhizome extracts showing more pronounced effects at the histological and molecular levels.

In the absence of detectable ptaquiloside, fiddleheads did not induce marked alterations in the evaluated parameters. In contrast, rhizome extracts were associated with more evident biological changes, suggesting the contribution of other bioactive compounds, such as pteroside derivatives.

These findings highlight the importance of considering the plant part, preparation method, route of exposure, and duration of intake when evaluating the biological effects and potential risks associated with *Pteridium aquilinum*.

## Figures and Tables

**Table 1:** Tentative assignment of the major compounds detected in the rhizome extract of *Pteridium aquilinum* by UHPLC-ESI-MS^n^.

Peak	RT (min)	UV	[M-H]^−^ (*m*/*z*)	MS/MS Fragments	Tentative Assignment
1	1.4	265, 374	133	MS^2^[133] = 115, 71, 87	Malic acid
2	1.8	264	191	MS^2^[191] = 111, 173, 129	Citric acid
3	7.6	289	341	MS^2^[341] = 179, 135	Caffeic acid glucoside
4	12.0	218, 261, 299	441	MS^2^[441] = 179, 395, 161, 143, 131	Pteroside C, P or M
5	12.6	227, 290	433	MS^2^[433] = 313, 343, 415	Naringenin-C-hexoside
6	13.2	232sh, 253, 290, 300sh, 331sh	513	MS^2^[513] = 469, 295, 269, 313, 339 MS^3^[513 → 469] = 295, 269, 313, 159, 199	Brainic acid
7	13.6	220, 263, 306	455	MS^2^[455] = 379, 409, 179, 161, 143	Pteroside A or A2
8	13.7	235, 291, 327	519	MS^2^[519] = 475, 409, 179 MS^3^[519 → 475] = 313	Blechnic acid hexose
9	13.9	219, 264, 303	455	MS^2^[455] = 409, 391, 161	Pteroside D
10	16.1	217, 261, 304	425	MS^2^[425] = 179, 379, 161, 143	Pteroside X or W
11	17.3	219, 261, 304	465	MS^2^[465] = 285, 391, 405, 423, 259, 243, 447, 373, 329	Pteroside derivative

**Table 2:** Tentative assignment of the major compounds detected of freeze-dried fiddleheads of *Pteridium aquilinum* by UHPLC-ESI-MS^n^.

Peak	RT (min)	λmax (nm)	[M-H]^−^ (*m*/*z*)	ESI-MS Fragments (*m*/*z*)	Probable Compound
1	1.3	276	133	MS^2^[133] = 115, 71, 87	Malic acid
2	1.6	280, 235sh	191	MS^2^[191] = 111, 173, 129	Citric acid
3	9.9	325, 301sh	335	MS^2^[335] = 179, 135, 161, 291	Caffeoyl-shikimic acid
4	10.1	326, 300sh	335	MS^2^[335] = 179, 135, 161, 291	Caffeoyl-shikimic acid (isomer)
5	11.1	250, 289sh 303, 335sh	403 *	MS^2^[403] = 385, 241, 375, 269, 343, 323, 359	Blechnic acid
6	12.1	250, 290sh, 302, 336sh	403 *	MS^2^[403] = 359, 385, 269, 313, 291, 341, 193	Blechnic acid (isomer)
7	12.9	265, 345	447	MS^2^[447]: 285, 327, 255, 151 MS^3^[447 → 285]: 257, 267, 241, 229, 213, 197, 163	Kaempferol-hexoside
8	16.6	265, 345	533	MS^2^[533] = 489, 445, 515, 371	Kaempferol-malonylhexoside
9	15.7	225, 241 290, 321	365	MS^2^[365]: 229, 135, 161, 185 MS^3^[365 → 229]: 185, 100, 130	Caffeoyl-tryptofan
10	15.9	243, 291, 318	593	MS^2^[593]: 285, 447, 307, 257 MS^3^[593 → 285]: 257, 151, 241, 213, 267, 229	Kaempferol-rutinoside
11	16.8	266	465	MS^2^[465] = 235, 220, 327, 271, 447	Unknown
12	17.0	290, 310sh, 240sh	349	MS^2^[349] = 229, 186, 145, 305, 220	Coumaroyl-tryptophan
13	17.3	242, 290, 319	379	MS^2^[379] = 229, 175, 335, 186	Feruloyl-tryptophan

* [M+HCOOH]^−^.

**Table 3:** Body weight (g) at the beginning and at the end of the study, weight gain (%), average daily consumption of the food (g) and drink (mL) during the experimental trial (mean ± standard deviation).

Groups	Humane Endpoints	Body Weight		Weight Gain (%)	Average Daily Consumption	
		Initial (g)	Final (g)		Food (g)	Drink (mL)
Assay 1: extract of *Pteridium aquilinum* rhizomes						
G1 (WT, control)	0.00 ± 0.00	29.72 ± 2.10	29.39 ± 2.47	−1.16 ± 3.00	3.52	4.61
G2 (WT, 0.05 g/mL)	0.00 ± 0.00	29.41 ± 2.66	30.94 ± 3.16	5.16 ± 4.36 ^1^	3.65	6.84
G3 (HPV, control)	0.04 ± 0.69	28.31 ± 2.63	28.15 ± 3.04	−0.62 ± 3.75	4.35	7.40
G4 (HPV, 0.0125 g/mL)	0.04 ± 0.89	26.99 ± 2.37	26.52 ± 2.06	−1.66 ± 2.43	4.21	8.39
G5 (HPV, 0.025 g/mL)	0.12 ± 0.11	26.17 ± 3.77	25.39 ± 3.84	−2.92 ± 6.21	4.19	7.56
G6 (HPV, 0.05 g/mL)	0.10 ± 0.14	28.59 ± 3.57	27.30 ± 2.84	−4.28 ± 2.61	4.58	10.00
Assay 2: extract of freeze-dried fiddleheads of *Pteridium aquilinum*						
G1 (WT, control)	0.00 ± 0.00	26.04 ± 0.46	30.42 ± 3.02	16.73 ± 10.02	3.73	4.15
G2 (WT, 50% PTE)	0.00 ± 0.00	24.85 ± 2.37	27.37 ± 3.03	10.43 ± 10.91	4.80	4.58
G3 (HPV, control)	0.25 ± 0.16	25.09 ± 0.71	26.86 ± 1.19	7.05 ± 2.45	4.43	7.42
G4 (HPV, 12.5% PTE)	0.12 ± 0.12	26.40 ± 1.93	28.00 ± 1.96	6.13 ± 3.47	4.25	6.92
G5 (HPV, 25% PTE)	0.16 ± 0.22	26.14 ± 1.41	26.93 ± 1.56	3.06 ± 4.08	4.43	7.08
G6 (HPV, 50% PTE)	0.12 ± 0.12	25.55 ± 1.25	26.86 ± 0.95	5.23 ± 4.42	4.69	7.09

^1^ Statistically different from G6 (*p* < 0.05).

**Table 4:** Relative organ weights in milligram (mg)/gram (g) (mean ± standard deviation).

Groups	Heart	Lung	Spleen	Liver	Kidneys
Assay 1: extract of *Pteridium aquilinum* rhizomes					
G1 (WT, control)	5.00 ± 1.62	6.20 ± 1.10	4.44 ± 0.38	41.46 ± 3.93	6.06 ± 1.03
G2 (WT, 0.05 g/mL)	4.50 ± 0.72	6.18 ± 0.67	3.84 ± 0.46	41.04 ± 2.90 ^1^	5.90 ± 0.45
G3 (HPV, control)	4.66 ± 0.55	6.64 ± 1.26	6.72 ± 2.62	45.26 ± 5.42 ^1^	7.28 ± 1.11
G4 (HPV, 0.0125 g/mL)	4.86 ± 0.77	7.20 ± 0.70	5.94 ± 2.49	48.16 ± 5.71	6.62 ± 0.49
G5 (HPV, 0.025 g/mL)	4.48 ± 0.32	7.90 ± 2.02	7.70 ± 3.14	50.06 ± 3.06	6.96 ± 1.54
G6 (HPV, 0.05 g/mL)	5.40 ± 0.99	7.54 ± 0.83	7.28 ± 2.91	54.00 ± 3.48	7.28 ± 0.78
Assay 2: extract of freeze-dried fiddleheads of *Pteridium aquilinum*					
G1 (WT, control)	4.16 ± 0.34	7.25 ± 0.84	4.14 ± 0.82	43.87 ± 3.90 ^2^	5.13 ± 0.53 ^2^
G2 (WT, 50% PTE)	4.12 ± 0.57	6.67 ± 0.27	3.73 ± 0.32	43.25 ± 2.66	4.97 ± 0.28 ^1^
G3 (HPV, control)	4.47 ± 0.46	6.92 ± 0.57	7.09 ± 4.55	50.88 ± 3.70	6.22 ± 0.28
G4 (HPV, 12.5% PTE)	4.44 ± 0.42	6.23 ± 0.39	5.37 ± 0.52	48.19 ± 1.64	5.67 ± 0.54
G5 (HPV, 25% PTE)	4.55 ± 0.42	6.45 ± 0.32	5.30 ± 1.00	48.42 ± 4.27	5.57 ± 0.18
G6 (HPV, 50% PTE)	4.62 ± 0.59	7.07 ± 0.76	5.37 ± 0.46	46.03 ± 2.20	6.04 ± 0.42

^1^ Statistically different from G6 (*p* < 0.05). ^2^ Statistically different from G3 (*p* < 0.05).

**Table 5:** Hematological and biochemical parameters evaluated (mean ± standard deviation) of the aqueous extract of *Pteridium aquilinum* rhizomes.

Parameters/Groups	G1 WT Control	G2 WT 0.05 g/mL	G3 HPV control	G4 HPV 0.0125 g/mL	G5 HPV 0.025 g/mL	G6 HPV 0.05 g/mL
Hematocrit (%)	46.55 ± 1.64	48.78 ± 2.90	37.93 ± 15.33	47.88 ± 3.16	45.76 ± 2.92	42.06 ± 15.29
Hemoglobin (M/μL)	15.15 ± 0.44	15.76 ± 0.77	12.33 ± 4.69	15.30 ± 1.10	14.46 ± 1.21	13.44 ± 4.82
Neutrophils (K/d/μL)	0.980 ± 0.37	0.966 ± 0.40	1.97 ± 0.95	0.828 ± 0.33	1.30 ± 0.95	5.36 ± 9.39
Lymphocytes (K/μL)	2.03 ± 0.96	1.85 ± 1.08	0.805 ± 0.09	1.17 ± 0.72	1.12 ± 0.54	2.25 ± 1.17
Monocytes (K/μL)	0.043 ± 0.03	0.076 ± 0.06	0.058 ± 0.06	0.050 ± 0.02	0.112 ± 0.14	0.436 ± 0.82
Eosinophils (K/μL)	0.04 ± 0.04	0.06 ± 0.02	0.07 ± 0.09	0.03 ± 0.03	0.08 ± 0.06	0.14 ± 0.07
Basophils (K/μL)	0.03 ± 0.01	0.02 ± 0.01	0.02 ± 0.01	0.04 ± 0.02	0.03 ± 0.04	0.03 ± 0.04
Leukocytes (K/μL)	3.12 ± 1.18	2.97 ± 1.41	2.92 ± 1.13	2.12 ± 1.10	2.64 ± 1.28	8.31 ± 11.36
Platelets (K/μL)	649.00 ± 127.28	782.40 ± 131.51	841.30 ± 462.14	1187.00 ± 180.60	1085.20 ± 133.12	981.20 ± 241.25
Erythrocytes (M/μL)	9.99 ± 0.27	10.7 ± 0.56	8.01 ± 3.00	10.18 ± 0.50	9.63 ± 0.88	8.92 ± 3.26
Reticulocytes (K/μL)	507.40 ± 74.98	509.00 ± 93.71	480.70 ± 92.10	482.70 ± 81.62	561.4 ± 98.03	386.10 ± 102.20
MPV	8.13 ± 0.53	8.06 ± 0.46	8.25 ± 0.78	7.90 ± 0.24	8.10 ± 0.33	8.02 ± 0.23
PDW	7.03 ± 0.73	6.96 ± 0.33	6.60 ± 0.14	6.83 ± 0.22	6.48 ± 0.24	6.64 ±0.49
MCV	46.60 ± 1.11	45.68 ±1.23	46.58 ± 3.41	47.00 ± 1.02	47.62 ± 1.71	47.20 ± 0.78
MCH	15.18 ± 0.13	14.76 ± 0.25	15.25 ± 0.56	15.00 ± 0.45	15.04 ± 0.39	15.16 ± 0.25
MCHC	32.55 ± 0.61	32.32 ± 0.45	32.80 ± 1.69	31.95 ± 0.26	31.58 ± 0.56	32.10 ± 0.80
Glucose	215.60 ± 39.53	231.30 ± 55.81	204.40 ± 61.52	152.40 ± 45.51	157.60 ± 50.34	162.60 ± 30.24
Total proteins (g/dL)	5.00 ± 0.24	5.48 ± 0.30	5.30 ± 1.09	5.90 ± 0.42	5.78 ± 0.81	5.34 ± 0.38
Albumine (g/dL)	2.49 ± 1.03	2.82 ± 0.18	2.37 ± 1.07	3.11 ± 0.04	3.10 ± 0.31	2.84 ± 0.24
ALAT (U/L)	22.86 ± 10.89	28.72 ± 4.97	51.15 ± 31.32	33.60 ± 14.60	31.05 ± 4.74	34.80 ± 17.87

MPV—mean platelet volume (fL); PDW—platelet distribution width (fL); MCV—mean corpuscular volume (fL); MCH—mean corpuscular hemoglobin (pg); MCHC—mean corpuscular hemoglobin concentration (M/μL); ALAT—alanine aminotransferase.

**Table 6:** Hematological and biochemical parameters (mean ± standard deviation) of the freeze-dried fiddleheads of *Pteridium aquilinum* evaluated.

Parameters/Groups	G1 WT Control	G2 WT 50% PTE	G3 HPV Control	G4 HPV 12.5% PTE	G5 HPV 25% PTE	G6 HPV 50% PTE
Hematocrit (%)	48.10 ± 2.24	47.96 ± 1.61	46.30 ± 1.39	46.18 ± 2.62	45.16 ± 2.03	47.28 ± 1.40
Hemoglobin (M/μL)	15.06 ± 0.55	15.28 ± 0.61	14.44 ± 0.50	14.74 ± 0.67	14.56 ± 0.55	15.00 ± 0.42
Neutrophils (K/d/μL)	0.76 ± 0.58	1.01 ± 0.45	0.89 ± 0.38	1.16 ± 0.32	1.18 ± 0.37	1.64 ± 0.39
Lymphocytes (K/μL)	2.26 ± 0.41	2.29 ± 0.60	2.28 ± 0.68	1.84 ± 0.73	1.83 ± 0.36	2.90 ± 1.03
Monocytes (K/μL)	0.05 ± 0.03	0.07 ± 0.01	0.26 ± 0.36	0.10 ± 0.04	0.10 ± 0.06	0.11 ± 0.04
Eosinophils (K/μL)	0.07 ± 0.02	0.05 ± 0.01	0.12 ± 0.08	0.07 ± 0.02	0.10 ± 0.06	0.10 ± 0.05
Basophils (K/μL)	0.01 ± 0.01	0.01 ± 0.02	0.01 ± 0.01	0.01 ± 0.01	0.02 ± 0.01	0.01 ± 0.01
Leukocytes (K/μL)	3.15 ± 0.88	3.43 ± 0.95	3.56 ± 1.21	3.19 ± 0.85	3.23 ± 0.50	4.76 ± 1.36
Platelets (K/μL)	637.80 ± 279.58	535.20 ± 167.96	875.40 ± 389.96	947.60 ± 399.20	983.20 ± 163.18	823.80 ± 139.64
Erythrocytes (M/μL)	9.71 ± 0.58	9.60 ± 0.17	9.77 ± 0.13	9.64 ± 0.43	9.57 ± 0.33	9.62 ± 0.37
Reticulocytes (K/μL)	531.34 ± 159.65	418.80 ± 123.26	487.08 ± 124.11	402.94 ± 126.37	353.02 ± 88.48	385.74 ± 58.30
MPV	8.48 ± 0.18	8.32 ± 0.16	8.30 ± 0.22	8.40 ± 0.72	8.20 ± 0.16	8.42 ± 0.24
PDW	7.68 ± 0.27	7.38 ± 0.54	7.62 ± 0.41	7.50 ± 0.34	7.24 ± 0.21	7.86 ± 0.46
MCV	49.54 ± 1.10	49.88 ± 1.29	47.40 ± 1.57	47.88 ± 0.78	47.18 ± 0.94	49.20 ± 1.29
MCH	15.50 ± 0.47	15.90 ± 0.49	14.80 ± 0.58	15.30 ± 0.16	15.24 ± 0.17	15.60 ± 0.29
MCHC	31.32 ± 0.61	31.86 ± 0.26	31.18 ± 0.78	31.94 ± 0.64	32.24 ± 0.39	31.72 ± 0.33
Glucose	159.60 ± 32.49	144.60 ± 21.42	179.60 ± 38.23	150.00 ± 41.44	135.60 ± 30.42	156.60 ± 41.75
Total proteins (g/dL)	5.44 ± 0.36	5.52 ± 0.23	6.00 ± 0.80	5.92 ± 0.18	5.56 ± 0.26	5.72 ± 0.23
Albumine (g/dL)	3.52 ± 0.19	3.34 ± 0.33	3.18 ± 0.19	3.15 ± 0.22	3.25 ± 0.20	3.10 ± 0.19
ALAT (U/L)	35.28 ± 7.64	40.42 ± 24.08	54.30 ± 10.49	47.08 ± 9.30	50.04 ± 12.09	49.70 ± 21.65

MPV—mean platelet volume (fL); PDW—platelet distribution width (fL); MCV—mean corpuscular volume (fL); MCH—mean corpuscular hemoglobin (pg); MCHC—mean corpuscular hemoglobin concentration (M/μL); ALAT—alanine aminotransferase.

**Table 7:** Number of animals (%) with histological lesions in all experimental groups from aqueous extract from *Pteridium aquilinum* rhizomes.

Histological Lesions	G1 WT Control	G2 WT 0.05 g/mL	G3 HPV Control	G4 HPV 0.0125 g/mL	G5 HPV 0.025 g/mL	G6 HPV 0.05 g/mL
EAR PAVILION LESIONS						
HYPERPLASIA						
	0(0%) ^1^	0(0%) ^2^	3(60%)	4(80%)	5(100%)	4(80%)
DYSPLASIA						
	0(0%) ^1^	0(0%) ^2^	4(80%)	3(60%)	2(40%)	4(80%)
CARCINOMA						
Carcinoma in situ	0(0%)	0(0%)	1(20%)	0(0%)	0(0%)	1(20%)
Squamous cell carcinoma	0(0%)	0(0%)	1(20%)	0(0%)	2(40%)	0(0%)
Invasive carcinoma	0(0%)	0(0%)	0(0%)	1(20%)	0(0%)	1(20%)
OTHER LESIONS						
Papilloma	0(0%)	0(0%)	0(%)	2(40%)	2(40%)	0(0%)
Hyperkeratosis—Orthokeratosis	0(0%)	0(0%) ^2^	2(40%)	3(60%)	3(60%)	4(80%)
Hyperkeratosis—Parakeratosis	0(0%) ^1^	0(0%) ^2^	5(100%) ^3^	4(80%)	1(20%)	4(80%)
Ulcer	0(0%)	0(0%)	2(40%)	0(0%)	0(0%)	1(20%)
CHEST SKIN LESIONS						
HYPERPLASIA						
	0(0%)	0(0%) ^2^	2(40%)	3(60%)	2(40%)	5(100%)
DYSPLASIA						
	0(0%)	0(0%) ^2^	1(20%)	3(60%)	0(0%)	4(80%)
CARCINOMA						
Carcinoma in situ	0(0%)	0(0%)	0(0%)	0(0%)	0(0%)	0(0%)
Squamous cell carcinoma	0(0%)	0(0%)	1(20%)	0(0%)	2(40%)	1(20%)
Invasive carcinoma	0(0%)	0(0%)	0(0%)	0(0%)	0(0%)	0(0%)
OTHER LESIONS						
Papilloma	0(0%)	0(0%)	0(0%)	0(0%)	0(0%)	0(0%)
Hyperkeratosis—Orthokeratosis	0(0%)	0(0%) ^2^	0(%) ^2^	2(40%)	2(40%) ^2^	5(100%)
Hyperkeratosis—Parakeratosis	0(0%)	0(0%) ^2^	1(20%) ^2^	1(20%) ^2^	1(20%) ^2^	5(100%)
Ulcer	0(0%)	0(0%)	0(0%)	0(0%)	2(40%)	0(0%)
LIVER						
Extramedullary hematopoiesis	0(0%)	0(0%)	0(0%)	0(0%)	1(20%)	1(20%)
Mononuclear infiltrate	0(0%)	4(80%) ^2^	1(20%)	1(20%)	0(0%)	0(0%)
Subacute multifocal hepatitis	0(0%)	0(0%)	2(40%)	1(20%)	0(0%)	1(20%)
Necrosis	0(0%)	0(0%)	1(20%)	0(0%)	0(0%)	1(20%)
SPLEEN						
Lymphoid hyperplasia	0(0%)	1(20%)	2(40%)	2(40%)	0(0%)	0(0%)
Hemosiderosis	0(0%)	2(40%)	0(0%)	0(0%)	1(20%)	2(40%)

^1^ Significantly different from the G3 (*p* < 0.05). ^2^ Significantly different from the G6 (*p* < 0.05). ^3^ Significantly different from the G2 (*p* < 0.05).

**Table 8:** Number of animals (%) with histological lesions in all experimental groups from extract freeze-dried fiddleheads of *P. aquilinum*.

Histological Lesions	G1 WT Control	G2 WT 50% PTE	G3 HPV Control	G4 HPV 12.5% PTE	G5 HPV 25% PTE	G6 HPV 50% PTE
EAR PAVILION LESIONS						
HYPERPLASIA						
	0(0%)	1(20%)	3(60%)	3(60%)	4(80%)	2(20%)
DYSPLASIA						
	0(0%)	0(0%)	4(80%)	2(40%)	3(60%)	4(80%)
CARCINOMA						
	0(0%)	0(0%)	0(0%)	0(0%)	0(0%)	0(0%)
OTHER LESIONS						
Sebaceous hyperplasia	0(0%)	0(0%) ^1^	0(0%) ^1^	0(0%) ^1^	2(40%)	5(100%)
Papilloma	0(0%)	0(0%)	0(0%)	0(0%)	0(0%)	0(0%)
Hyperkeratosis—Parakeratotic	0(0%)	0(0%) ^1^	4(80%)	5(100%)	4(80%)	5(100%)
Inflammatory infiltrate	0(0%) ^2^	0(0%) ^1^	5(100%)	5(100%)	5(100%)	5(100%)
Erosion	0(0%)	0(0%)	0(0%)	3(60%)	0(0%)	0(0%)
Ulceration	0(0%)	0(0%)	1(20%)	0(0%)	0(0%)	1(20%)
Severe deformation	0(0%)	0(0%) ^1^	0(0%) ^1^	0(0%) ^1^	4(80%)	5(100%)
CHEST SKIN LESIONS						
Sebaceous hyperplasia	0(0%)	0(0%) ^1^	3(60%)	3(60%)	4(80%)	5(100%)
HYPERPLASIA						
	0(0%)	0(0%)	2(40%)	2(40%)	1(20%)	0(0%)
DYSPLASIA						
	0(0%)	0(0%)	1(20%)	1(20%)	0(0%)	0(0%)
CARCINOMA						
	0(0%)	0(0%)	0(0%)	0(0%)	0(0%)	0(0%)
OTHER LESIONS						
Inflammatory infiltrate	0(0%)	1(20%)	4(80%)	3(60%)	1(20%)	4(80%)
Cyst dermoid	0(0%)	0(0%)	0(0%)	1(20%)	0(0%)	0(0%)
LIVER						
Inflammatory Infiltrate	0(0%)	4(80%)	2(40%)	0(0%)	4(80%)	4(80%)
Tumefaction	0(0%)	0(0%)	0(0%)	1(20%)	2(40%)	1(20%)
Hydropic degeneration	0(0%)	0(0%)	0(0%)	0(0%)	0(0%)	0(0%)
SPLEEN						
Hyperemia	0(0%)	0(0%)	0(0%)	0(0%)	0(0%)	0(0%)
Inflammatory infiltrate	0(0%)	0(0%)	2(40%)	5(100%)	4(80%)	2(40%)
Mesangial expansion	0(0%)	0(0%)	0(0%)	0(0%)	0(0%)	0(0%)
Hydronephrosis	0(0%)	0(0%)	0(0%)	0(0%)	0(0%)	0(0%)

^1^ Significantly different from the G6 (HPV, 50% PTE) (*p* < 0.05). ^2^ Significantly different from the G3 (HPV, control) (*p* < 0.05).

## Data Availability

Data will be made available on request.

## Supplementary Materials

The following supporting information can be downloaded at: https://www.mdpi.com/article/10.3390/biology15120976/s1, Figure S1: Chromatographic profile of *Pteridium aquilinum* rhizomes extract recorded at 280 nm; Figure S2: Chromatographic profile of freeze-dried fiddleheads of *Pteridum aquilinum*, recorded at 280 nm; Table S1. Tentatively assignment of the major compounds detected in the rhizomes extract of *Pteridium aquilinum* by UHPLC-ESI-MS^n^. Table S2: Tentatively assignment of the major compounds detected of freeze-dried fiddleheads of *Pteridium aquilinum* by UHPLC-ESI-MS^n^.
