# Supplementary material for: Toxicological Activities of Pteridium aquilinum Rhizomes and Fiddleheads in HPV16-Transgenic Mice
Source: Biology (Basel). 2026 Jun 22;15(12):976. doi: 10.3390/biology15120976 (PMC13296977; doi:10.3390/biology15120976)
Supplement: Supplementary file 1 [file biology-15-00976-s001.zip › biology-4339857-supplementary.pdf]

Supplementary Section

The UHPLC-ESI-MS analysis of the rhizome extract allowed us to conclude that it is particularly rich in pteroside derivatives (**supplementary Figure S1 and supplementary Table S1**). These compounds eluted in peaks 4, 7, 9, 10 and 11, mostly in the form of COOH adducts, and presented a UV spectrum typical of pterosins, with three points of maximum absorption at approximately 220, 260 and 300 nm. Additionally, all of them generated product ions at m/z 179 and 161, as well as neutral losses of 180 and 162 Da, characteristic of the fragmentation of glycosidic bonds. The product ion at m/z 395 (loss of COOH) appearing at the MS/MS spectrum of the compound eluting at 12.0 min ([M – H]- at m/z 441) indicated the presence of one of the three pteroside isomers, namely pteroside C, P or M (33–35). The compound eluting in peak 10 was identified as pteroside X or W due to the appearance of the product ion at m/z 379, which is indicative of pterosin X or W, i.e., the respective aglycones after losing the sugar residue [36, 37]. Interestingly, two compounds with the same [M – H]- at m/z at 455 eluted at different times, i.e., at 13.6 and 13.9 min. The product ion at m/z 409 in both MS/MS spectra together with the neutral losses of 248 (pterosin A moiety) and 230 (dehydroxylated pterosin A moiety) indicate that these compounds correspond the isomers pteroside A, pteroside A2 or pteroside D. A slight difference can be noticed, however, in the MS/MS fragmentation pattern of these two compounds, the former presenting as a major product ion at m/z 379, which is not visible in the second compound.

This product ion results from the neutral loss of 30 Da and may indicate that this compound is a pteroside A since the backbone of this compound contains a hydroxymethyl group in carbon 2 of the pterosin moiety that can break more easily than the glycosidic bond, thus originating this pattern [38]. Pteroside A2 could also originate this product ion due to the presence of the hydroxyethyl group in carbon 6 of its pterosin moiety [38]. In turn, none of these features are present in the backbone of the pterosin moiety of pteroside D. Instead, it contains a hydroxyl group in carbon 3, which, upon fragmentation, could be lost and give rise to the product ion observed at m/z 391 [39]. Following this logic, the compound eluting in peak 7 was assigned to pteroside A or A2, while that in peak 9 was tentatively identified as pteroside D. Finally, although it was not possible to properly identify the compound eluting in peak 11, its UV spectrum highly resembles the spectrum of a pteroside and its main product ion at m/z 285 resulted in from the neutral loss of 180 Da, i.e., a sugar residue. Therefore, it is highly possible that this compound corresponds to a pteroside derivative. Apart from the pteroside compounds, two phenolic compounds were detected in the aqueous extract of P. aquilinum rhizomes, eluting in peaks 3 and 5. The former presented an UV and MS spectra typical of caffeic acid-O-glucoside (UVmax at 289 nm and [M – H]- at m/z at 341 fragmenting into product ions at m/z 179 and 135) [40], while the latter showed a high resemblance to what has been previously described for naringenin-C-hexose (UVmax at 290 nm and [M – H]- at m/z at 433, fragmenting into product ions at m/z 313, 343 and 415) [40]. Additionally, two dihydrobenzofuran neolignanes were found eluting in peaks 5 and 8 and assigned to brainic acid and blechnic acid hexoside, based on their MS profile coherent with previously reported data, as well as their UV spectra [41]. Interestingly, although blechnic acid derivatives have been commonly reported in other ferns, this type of compound is usually characteristic of species belonging to the Blechnaceae family [42] and has never been described in other species before.

In turn, the phytochemicals detected in the *Pteridium aquilinum* fiddlehead samples (**supplementary Figure S2 and supplementary Table S2)** were predominantly phenolic compounds. Among them, two caffeoyl-shikimate isomers were detected in peaks 3 and 4, demonstrating a UV spectrum and MS fragmentation pattern coherent with what has been described before [43]. Two isomers of blechnic acid (an ester derivative of caffeic acid) were detected in their aglycone form in the fiddlehead sample as well, eluting in peaks 5 and 6. These compounds both appeared in the MS spectrum as a formate adduct [M + HCOO]- and revealed a UV spectrum matching what has been reported in Wada et al.'s studies [41]. Apart from these, two tryptophan derivatives of caffeic acid were detected eluting in peak 9 (caffeoyl-tryptophan) and 13 (feruloyl-tryptophan), both showing neutral losses of 204 Da in the MS/MS spectrum, which indicates the loss of a tryptophan moiety [44, 45]. Another tryptophan-phenolic derivative was found in peak 12, corresponding to coumaroyl tryptophan, which also showed the common loss of 204 Da characteristic of the amino acid [44].

In addition, the detected flavonoids were all kaempferol derivatives. These eluted in peaks 7, 8 and 10, corresponding to kaempferol-hexoside, kaempferol malonylhexoside and kaempferol rutinoside, respectively, all presenting the same main product ion at m/z 285, which, allied to the UV spectrum, is indicative of a kaempferol aglycone [46] [45].

Interestingly, no ptaquiloside or any of its derivatives was detected in this sample. The washing step combined with the solvent used for the fiddlehead extraction could explain the absence of these compounds, since they are known to be very soluble in water [47]; therefore, a good part of them may have been removed with the sample washing, and ethanol 80% may not be very effective for extracting the possible residual concentrations that may have been left afterwards.

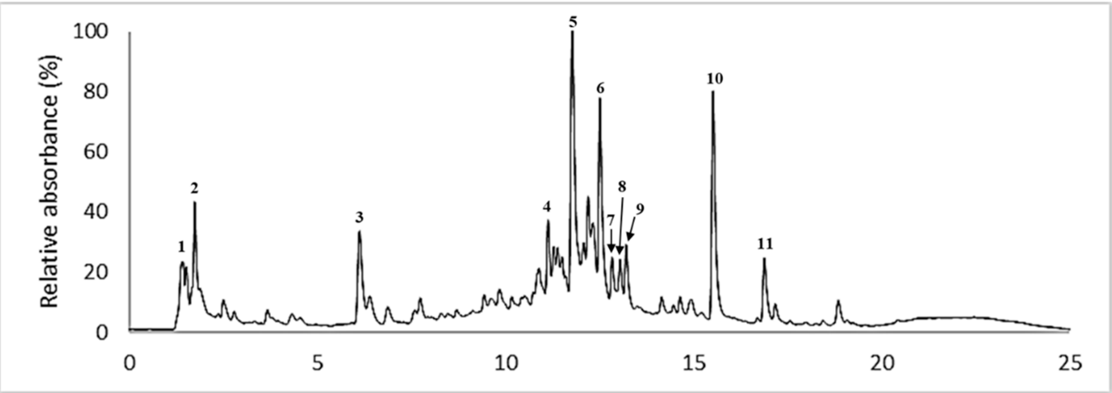

Supplementary Figure S1. Chromatographic profile of *Pteridium aquilinum* rhizomes extract recorded at 280 nm. Numbers in the figure correspond to the UHPLC-ESI-MSn peaks listed in supplementary Table S1.

Supplementary table S1. Tentatively assignment of the major compounds detected in the rhizomes extract of *Pteridium aquilinum* by UHPLC-ESI-MS^n.

Peak	RT (min)	UV	[M – H]- (m/z)	MS/MS fragments	Tentative assignment
**1**	1.4	265, 374	133	MS2[133] = 115, 71, 87	Malic acid
**2**	1.8	264	191	MS2[191] = 111, 173, 129	Citric acid
**3**	7.6	289	341	MS2[341] = 179, 135	Caffeic acid glucoside
**4**	12.0	218, 261, 299	441	MS2[441] = 179, 395, 161, 143, 131	Pteroside C, P or M
**5**	12.6	227, 290	433	MS2[433] = 313, 343, 415	Naringenin-C-hexoside
**6**	13.2	232sh, 253, 290, 300sh, 331sh	513	MS2[513] = 469, 295, 269, 313, 339<br>MS3[513→469] = 295, 269, 313, 159, 199	Brainic acid
**7**	13.6	220, 263, 306	455	MS2[455] = 379, 409, 179, 161, 143	Pteroside A or A2
**8**	13.7	235, 291, 327	519	MS2[519] = 475, 409, 179<br>MS3[519→475] = 313	Blechnic acid hexose
**9**	13.9	219, 264, 303	455	MS2[455] = 409, 391, 161	Pteroside D
**10**	16.1	217, 261, 304	425	MS2[425] = 179, 379, 161, 143	Pteroside X or W
**11**	17.3	219, 261, 304	465	MS2[465] = 285, 391, 405, 423, 259, 243, 447, 373, 329	Pteroside derivative

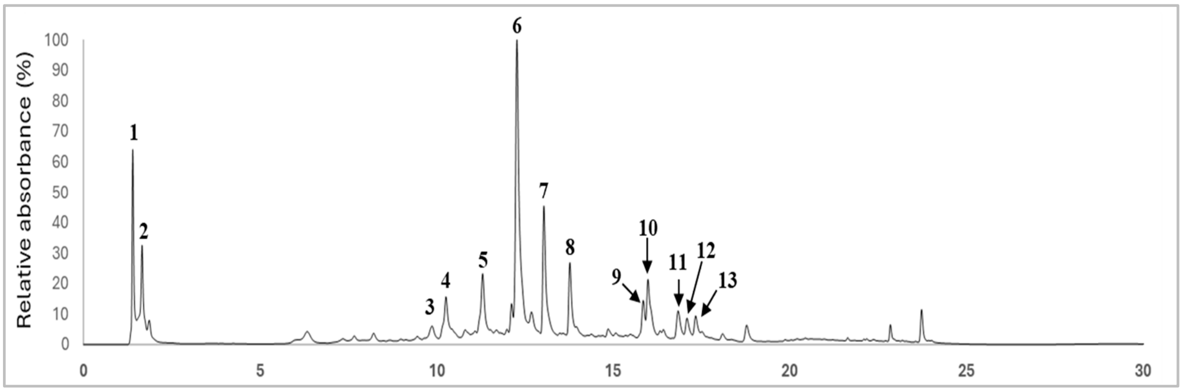

Supplementary Figure S2. Chromatographic profile of freeze-dried fiddleheads of *Pteridum aquilinum,* recorded at 280 nm. Numbers in the figure correspond to the UHPLC-ESI-MSn peaks listed in supplementary Table S2.

Supplementary Table S2. Tentatively assignment of the major compounds detected of freeze-dried fiddleheads of *Pteridium aquilinum* by UHPLC-ESI-MS^n.

Peak	RT (min)	λmax (nm)	$[M-H]^-$ (m/z)	ESI-MS fragments (m/z)	Probable compound
**1**	1.3	276	133	MS^2[133] = 115, 71, 87	Malic acid
**2**	1.6	280, 235sh	191	MS^2[191] = 111, 173, 129	Citric acid
**3**	9.9	325, 301sh	335	MS^2[335] = 179, 135, 161, 291	Caffeoyl-shikimic acid
**4**	10.1	326, 300sh	335	MS^2[335] = 179, 135, 161, 291	Caffeoyl-shikimic acid (isomer)
**5**	11.1	250, 289sh 303, 335sh	403*	MS^2[403] = 385, 241, 375, 269, 343, 323, 359	Blechnic acid
**6**	12.1	250, 290sh, 302, 336sh	403*	MS^2[403] = 359, 385, 269, 313, 291, 341, 193	Blechnic acid (isomer)
**7**	12.9	265, 345	447	MS^2 [447]: 285, 327, 255, 151<br>MS3 [447→285]: 257, 267, 241, 229, 213, 197, 163	Kaempferol-hexoside
**8**	16.6	265, 345	533	MS^2[533] = 489, 445, 515, 371	Kaempferol-malonylhexoside
**9**	15.7	225, 241 290, 321	365	MS^2 [365]: 229, 135, 161, 185<br>MS^3 [365→229]: 185, 100, 130	Caffeoyl-tryptofan
**10**	15.9	243, 291, 318	593	MS^2 [593]: 285, 447, 307, 257<br>MS^3 [593→285]: 257, 151, 241, 213, 267, 229	Kaempferol-rutinoside
**11**	16.8	266	465	MS^2[465] = 235, 220, 327, 271, 447	Unknown
**12**	17.0	290, 310sh, 240sh	349	MS^2[349] = 229, 186, 145, 305, 220	Coumaroyl-tryptophan
**13**	17.3	242, 290, 319	379	MS^2[379] = 229, 175, 335, 186	Feruloyl-tryptophan

* [M+HCOOH]-
